# Current Research in Drug-Free Cancer Therapies

**DOI:** 10.3390/bioengineering12040341

**Published:** 2025-03-26

**Authors:** Akshaya Andavar, Varsha Rajesh Bhagavathi, Justine Cousin, Nirvi Parekh, Zahra Sadat Razavi, Bo Tan

**Affiliations:** 1 Karpagam Academy of Higher Education, Coimbatore 641021, India; akshayaandavar32@gmail.com; 2 SRM Easwari Engineering College, Chennai 600089, India; 3 École Publique d’Ingénieurs de la Santé et du Numérique (EPISEN), Université Paris-Est Créteil (UPEC), 94000 Créteil, France; justine.cousin92@gmail.com; 4 Institute of Chemical Technology, Nathalal Parekh Marg, Matunga, Mumbai 400019, India; nirvi.parekh.2003@gmail.com; 5 Physiology Research Center, Iran University Medical Sciences, Tehran 1416634793, Iran; razavis93@gmail.com; 6 Institute of Biomedical Engineering Science and Technology (iBEST), Faculty of Engineering and Architectural Science, Toronto Metropolitan University, Toronto, ON M5B 2K3, Canada

**Keywords:** drug-free therapy, cancer treatment, nanotechnology, nanoparticles, immunotherapy, personalized medicine

## Abstract

Cancer treatment has historically depended on conventional methods like chemotherapy, radiation, and surgery; however, these strategies frequently present considerable limitations, including toxicity, resistance, and negative impacts on healthy tissues. In addressing these challenges, drug-free cancer therapies have developed as viable alternatives, utilizing advanced physical and biological methods to specifically target tumor cells while reducing damage to normal tissues. This review examines several drug-free cancer treatment strategies, such as high-intensity focused energy beams, nanosecond pulsed electric fields, and photothermal therapy as well as the use of inorganic nanoparticles to promote selective apoptosis. We also investigate the significance of targeting the tumor microenvironment, precision medicine, and immunotherapy in the progression of personalized cancer therapies. Although these approaches demonstrate significant promise, challenges including scalability, safety, and regulatory obstacles must be resolved for clinical application. This paper presents an overview of current research in drug-free cancer therapies, emphasizing recent advancements, underlying scientific principles, and the steps required for clinical implementation.

## 1. Introduction

Cancer remains one of the most complex and devastating diseases afflicting humanity, with millions of new cases diagnosed each year worldwide. The traditional approaches to cancer treatment, including chemotherapy, radiation therapy, and surgical interventions, have long been the cornerstone of oncological care. While these treatments have significantly improved patient survival rates and provided effective means of tumor reduction, they are often accompanied by severe side effects, including systemic toxicity, immunosuppression, and multidrug resistance. The limitations of conventional therapies have driven the scientific community to explore alternative and innovative approaches that can effectively combat cancer while minimizing harm to normal cells and enhancing patient quality of life [1]. Among these emerging strategies, drug-free cancer therapies have garnered significant attention as promising, non-toxic interventions aimed at selectively targeting cancer cells while preserving surrounding healthy tissues. The concept of drug-free cancer therapy encompasses a broad spectrum of techniques that leverage physical, biological, and technological advancements to eliminate cancer cells without relying on conventional pharmacological agents. In the context of cancer treatment, the term “drug-free” describes treatment approaches devoid of traditional pharmacological agents, such as targeted drug therapy or chemotherapy, to generate their therapeutic effects. Rather, these methods minimize systemic toxicity by means of physical forces, biological mechanisms, or technical breakthroughs, therefore inducing cancer cell death selectively [2]. Energy-based treatments including high-intensity focused ultrasonic (HIFU), nanosecond pulsed electric fields (nsPEFs), and photothermal ablation as well as nanoparticle-mediated interventions dependent on external stimuli such heat, light, or mechanical forces constitute part of drug-free therapies. Unlike pharmacological treatments, which sometimes involve systemic drug administration leading to widespread toxicity, these non-pharmacological methods maximize the unique biophysical features of cancer cells, such as changed membrane permeability and metabolic vulnerabilities, to achieve targeted destruction. With the possibility to overcome medication resistance, limit unwanted effects, and improve patient quality of life, this basic difference emphasizes the justification for drug-free cancer therapy as a hopeful substitute for conventional treatments [3]. Although these treatments use advanced biomedical technology to attain therapeutic efficacy, the lack of conventional chemotherapeutic medications emphasizes their classification as “drug-free”. By customizing treatments to the particular genetic, molecular, and cellular features of a malignancy, precision medicine sets itself apart from traditional pharmacological drugs [4]. Many times, as they are broad-spectrum and meant to target fast-dividing cells, traditional pharmacological therapies including chemotherapy can cause significant collateral damage to healthy tissues. By means of molecular diagnostics, biomarker profiling, and advanced imaging technologies to create individualized treatment strategies, precision medicine strives to maximize therapeutic efficacy. This covers non-drug treatments including immunotherapy, gene editing, and tumor microenvironment modification in addition to focused medication therapies. Because traditional medicine, especially herbal and holistic approaches, depends on natural chemicals and lifestyle changes rather than synthetic medications, it is often regarded as “drug-free”. Although certain ancient medicines include pharmacological effects from bioactive substances, their complicated, multi-component character and lack of regular dosage make them usually not categorized under conventional drug-based treatments [5]. Moreover, traditional pharmacological agents including small-molecule inhibitors and chemotherapy operate through well-defined biochemical pathways, usually requiring systemic delivery and metabolism, which could cause side effects like toxicity and drug resistance [1]. Clarifying the differences between these therapeutic modalities’ conventional pharmacology, precision medicine, and traditional medicine helps contextualize the expanding interest in drug-free cancer therapy as a new, integrative strategy in oncological care. These strategies include high-intensity focused energy beams, such as ultrasound, electric pulses, and photothermal ablation, as well as the use of engineered nanoparticles and nanobubbles to induce targeted cellular destruction. Unlike traditional treatments, which often involve systemic drug administration leading to widespread toxicity, drug-free cancer therapies seek to harness intrinsic cellular vulnerabilities, such as sensitivity to thermal, mechanical, or electrical stimuli, to induce apoptosis or necrosis selectively. This novel approach not only mitigates adverse side effects but also reduces the risk of drug resistance, a significant challenge in long-term cancer management [6]. A key component of drug-free cancer therapy is the utilization of high-intensity focused energy beams, including nanosecond pulsed electric fields (nsPEFs), high-intensity focused ultrasound (HIFU), and laser-induced photothermal therapy. These modalities exploit the unique properties of cancer cells, such as altered dielectric constants, increased membrane permeability, and metabolic abnormalities, to induce localized cellular destruction [7,8]. For instance, nsPEFs disrupt intracellular signaling pathways and induce apoptosis without compromising plasma membrane integrity, thereby ensuring targeted tumor eradication with minimal collateral damage. Similarly, HIFU and photothermal therapy leverage acoustic or electromagnetic energy to generate localized hyperthermia, effectively coagulating and destroying tumor tissues while sparing surrounding structures. The precise and non-invasive nature of these techniques makes them particularly advantageous for treating deep-seated and surgically inaccessible tumors [9,10]. Another groundbreaking advancement in drug-free cancer therapy is the use of nascent inorganic nanoparticles to induce selective tumor cell apoptosis. Recent research has demonstrated that engineered nanoparticles, such as gold nano shells, carbon nanotubes, and quantum dots, exhibit unique physicochemical properties that enable their application in cancer theragnostic. These nanoparticles can be functionalized to enhance their biocompatibility, allowing for their targeted accumulation in tumor tissues through passive and active mechanisms. Upon external stimulation such as near-infrared (NIR) irradiation, ultrasound waves, or magnetic fields, these nanoparticles can generate heat, reactive oxygen species (ROS), or mechanical disruption, thereby triggering cancer cell death through non-pharmacological means. Furthermore, nanoparticle-based therapies offer the potential for real-time imaging and monitoring, enabling clinicians to assess treatment efficacy and tailor interventions with unprecedented precision [11,12]. In addition to directly attaching on tumors, drug-free cancer therapies also encompass approaches that modulate the tumor microenvironment to enhance immune recognition and suppression of malignant cells. Techniques such as ultrasound-mediated immunomodulation, tumor electrostimulation, and epigenetic reprogramming via engineered nanoparticles aim to alter the biochemical landscape of tumors, making them more susceptible to immune clearance. Moreover, targeting the tumor microenvironment has become a critical component of precision medicine, ensuring that treatments are tailored to the specific cellular and molecular characteristics of each patient’s cancer [13]. The integration of immunotherapy into drug-free cancer treatments has shown immense potential in harnessing the body’s natural immune defenses to combat tumors more effectively. Strategies such as checkpoint inhibitors, adoptive T-cell therapy, and cancer vaccines are being explored in conjunction with drug-free modalities to develop more comprehensive and personalized cancer treatments [14]. Despite the tremendous promise of drug-free cancer therapies, several challenges must be addressed before their widespread clinical implementation. The scalability and reproducibility of nanomaterial synthesis, optimization of energy delivery parameters, and long-term safety assessments remain critical areas of ongoing investigation. Additionally, regulatory frameworks for nanomedicine and non-drug interventions must evolve to accommodate these novel treatment paradigms, ensuring rigorous evaluation of their therapeutic benefits and potential risks. Collaborative efforts between interdisciplinary research teams, industry stakeholders, and regulatory bodies are essential to accelerate the translation of these technologies from bench to bedside. As we continue to push the boundaries of cancer treatment, the advent of drug-free cancer therapies represents a paradigm shift in oncological care [15,16]. By capitalizing on the principles of physics, nanotechnology, and bioengineering, these approaches offer a promising alternative to conventional chemotherapy and radiation, addressing the unmet needs of patients seeking safer, more effective treatment options. The integration of non-pharmacological strategies with existing therapeutic modalities holds the potential to revolutionize cancer care, paving the way for a future where precision-targeted, minimally invasive interventions become the new standard in oncology. With continued research and technological refinement, drug-free cancer therapies may ultimately redefine the landscape of cancer treatment, bringing us closer to a world where cancer is not only manageable but curable without the burden of systemic toxicity [17,18,19,20]. This review aims to provide a comprehensive overview of the current state of research in drug-free cancer therapies, focusing on their scientific principles, recent advancements, and the challenges that must be overcome for clinical translation. Additionally, we explore the role of tumor microenvironment targeting, precision medicine, and immunotherapy in developing more effective, personalized cancer treatments. By synthesizing current knowledge and identifying future directions, this review seeks to contribute to the ongoing efforts in transforming cancer therapy into a more patient-centric, minimally invasive, and highly effective discipline.

## 2. High-Intensity Focused Energy Beams for Cancer Therapy

### 2.1. Apoptosis Induction with Nanosecond Pulsed Electric Fields

**Figure 1:**
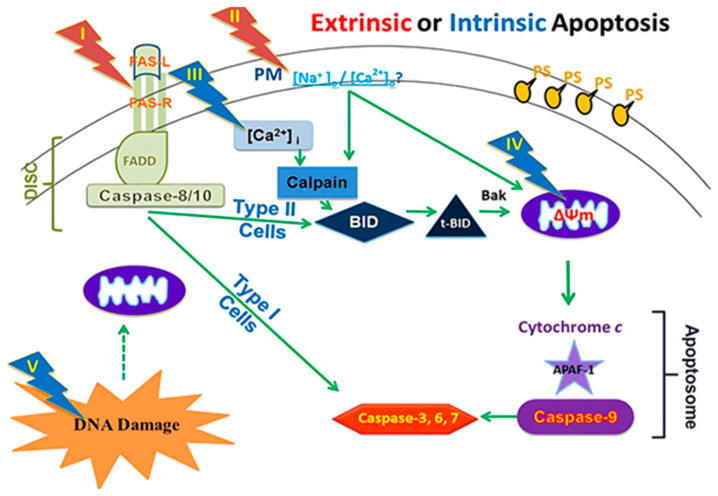
A framework for assessing nsPEFs impacts on cellular targets and apoptosis mechanisms [22].

The induction of apoptosis (programmed cell death) through electric pulses represents an innovative approach to cancer treatment without the use of cytotoxic drugs. This method involves the application of nanosecond pulsed electric fields (nsPEFs) directly to cancer cells. This approach is a part of new cancer therapies aimed at minimizing the side effects of pharmacological treatments while maximizing efficacy against tumor cells (Figure 1) [21].

The effects of nsPEFs on cells are parameter-dependent; the application of high-intensity electric fields (in the order of MV/cm) for several tens of nanoseconds can affect intracellular signal transduction and cell structures while maintaining plasma membrane integrity. Pulses of 10 ns at 100 kV/cm cause intracellular changes without damaging the cell membrane, while pulses of 100 μs at 1 kV/cm cause reversible electroporation without affecting cell function [23]. Studies show that intermediate parameters can induce significant apoptosis in vitro in several types of cancer cells, a process called apoptosis induction by electric pulses (AIEP), which is irreversible. This apoptosis also depends on pulse duration, with longer pulses (around 300 ns) showing stronger apoptosis markers than shorter pulses (around 60 ns) and very short pulses (10 ns) showing negligible effects. Cell responses vary according to cell type [24]. The efficacy of electric pulses in inhibiting cancer cells has been demonstrated by numerous experiments. In vitro studies revealed significant apoptosis in human ovarian carcinoma cells (SKOV3) after treatment with 10 kV/cm electric pulses during 100 ns3-5. Pulsed electric fields above 21 kV/cm for 300 ns can cause rapid shrinking of tumor cell nuclei. The application of 40 kV/cm electric pulses for 300 ns on murine melanomas in vivo led to complete tumor remission. Cancer cells showed a higher apoptosis rate when exposed to the same electric field as normal cells due to their higher dielectric constant and larger nucleus [25]. The plasma membrane is essential for cell survival. If it is damaged or disrupted, it can trigger internal processes leading to cell death. Conventional electroporation uses electric pulses to create pores in the plasma membrane, allowing substances to enter the cell but potentially damaging the membrane. The application of microsecond or longer-duration electric fields to cells causes large accumulations of oppositely charged ions on either side of the cell surface. If too much charge accumulates at the cell membrane, the electric field breaks the membrane. Large holes (or pores) form in the membrane, allowing ions to spill out [26]. This effect is called electroporation. When electroporation is applied only to the membranes of organelles inside the cell without affecting the plasma membrane, it is referred to as intracellular electro manipulation. Electric pulses create pores in the plasma membrane, and the more numerous or larger these pores are, the higher the probability of triggering apoptosis, and the extent of this damage determines the degree of apoptosis. Electric pulses can also destroy lymphatic capillaries around cancer tissue, reducing the possibility of lymphatic metastasis after treatment. They can decrease blood flow to the tumor and reduce the expression of vascular endothelial growth factor (VEGF) in vessels near cancer tissues, limiting metastatic growth [27].

**Figure 2:**
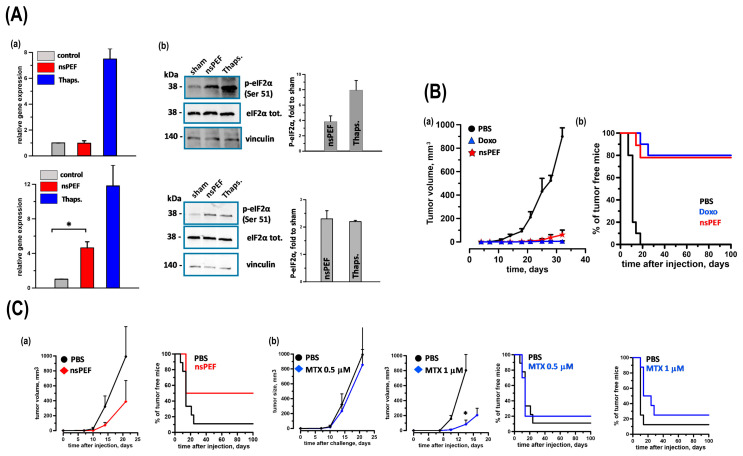
(**A**) **Activation of ER Stress Sensors IRE1 and PERK.** EL-4 and CT26 cells were treated with iso-effective doses of nsPEFs (100 and 300 pulses, respectively; 200 ns, 7 kV/cm, 10 Hz). Samples were collected 5 h post treatment. IRE1 Activation (**a**): XBP1 expression in EL-4 and CT26 cells was quantified using real-time PCR, normalized to HPRT mRNA, and expressed as relative expression levels. PERK Activation (**b**): Phosphorylation of eIF2α was assessed by Western blot with anti-phospho-eIF2α (Serine 51) antibody. Representative blots for EL-4 and CT26 cells showed total and phosphorylated eIF2α, with Vinculin as a loading control. Quantifications were expressed as fold change relative to sham treatment. Thapsigargin (1 μM) served as a positive control. Mean ± s.e.; n = 3 for both assays. *p* < 0.001 *for nsPEFs* vs. *sham.* * *p* < 0.01. (**B**) **Tumor Immunogenicity of nsPEFs-treated CT26 Cells** CT26 tumor cells were subjected to nsPEFs (600 pulses, 200 ns, 7 kV/cm, 10 Hz) and immediately injected into the flank of syngeneic BalbC mice (0.6 × 10^6^ cells/mouse). Control groups received either PBS or CT26 cells treated with doxorubicin (Doxo, 25 μM, 24 h). Seven days later, animals were challenged with live tumor cells (0.1 × 10^6^ cells/mouse) in the opposite flank. Tumor Growth and Protection: Panel (**a**) shows tumor growth curves, while (**b**) presents the percentage of tumor-free animals. Data represent animals without tumor development at the vaccination site. Mean ± s.e.; n = 10, 10, and 9 for PBS, Doxo, and nsPEFs groups, respectively. (**C**) **Comparative Immunogenicity of nsPEFs and Mitoxantrone-treated EL-4 Cells EL-4** tumor cells were treated with nsPEFs (200 pulses, 200 ns, 7 kV/cm, 10 Hz) or mitoxantrone (MTX, 0.5 μM or 1 μM, 24 h) and injected into syngeneic C57BL6 mice (0.6 × 10^6^ cells/mouse). Control groups received PBS. Seven days post vaccination, animals were challenged with live EL-4 cells (0.03 × 10^6^ cells/mouse) in the opposite flank. Tumor Growth and Protection: Top graphs show tumor growth curves, and bottom graphs display the percentage of tumor-free animals. Data represent animals without tumor development at the vaccination site. Mean ± s.e.; n = 9 (PBS) and 6 (nsPEFs) in (**a**) and n = 9, 9, 8, and 8 for PBS, 0.5 μM MTX, PBS, and 1 μM MTX groups in (**b**), respectively. *p <* 0.01 [29].

Multiple cancer cell lines have been studied to explore their potential applications in cancer therapy, including liver cancer cells (SMMC7721 and HepG2), human leukemia cells (Jurkat and HL-60), and tongue squamous cell carcinoma cells (SCC-9). Studies have also been conducted on other types of cancer, such as melanoma and colon carcinoma. Research has extended to the use of murine models, particularly immunodeficient mice, to study the effectiveness of nsPEFs in reducing tumor size and inhibiting tumor progression. However, some studies have been limited to in vitro experiments without explicit mention of animal model usage. Cancer cell death can be immunogenic or non-immunogenic depending on the stimulation. Immunogenic cell death (ICD) inducers use endoplasmic reticulum (ER) stress to traffic danger signals like CRT and ATP [28]. Rossi et al. discovered that nanosecond pulsed electric fields (nsPEFs), a new tumor ablation method, activate the ER-resident stress sensor PERK in CT-26 colon cancer and EL-4 lymphoma cells. Apoptosis induction and persistent CRT exposure on the plasma membrane are associated with PERK activation in both nsPEFs-treated cell types. Caspase-3/7 activity rose fourteen-fold in CT-26 cells and four-fold in EL-4 cells [29]. Moreover, nsPEFs treatments released HMGB1 in both cell lines, although only CT-26 showed extracellular ATP. Finally, in vaccination experiments, CT-26 cells treated with nsPEFs or doxorubicin equally inhibited tumor development at challenge sites, producing a protective anticancer immune response in 78% and 80% of animals. Though less effective than CT-26, nsPEFs- and mitoxantrone-treated EL-4 cells saved 50% and 20% of rats, respectively. These findings confirm the notion that nsPEFs cause ER stress and true ICD (Figure 2).

There have been no human experiments yet, but it is anticipated that electric pulses with defined parameters could be applied without significant side effects. AIEP offers advantages for cancer treatment without cytotoxic drugs, allowing selective destruction of tumor cells with low energy input, thus minimizing damage to surrounding healthy tissues. The clinical preparation of these technologies involves the development of medical devices capable of safely and effectively delivering nsPEFs in humans. This also requires extensive preclinical studies to confirm their safety and efficacy, followed by the organization of clinical trials [30]. Nanosecond pulsed electric fields (nsPEFs) represent a novel and potentially revolutionary approach to cancer treatment, offering significant benefits while also presenting challenges and risks. Among the advantages, nsPEFs enable the targeting and elimination of tumors in a non-thermal manner, thereby preserving adjacent healthy tissues and minimizing side effects such as scarring and inflammation. Additionally, nsPEFs can reduce tumor angiogenesis and potentially induce an anticancer immune response, thus lowering the risk of recurrence. Furthermore, nsPEFs demonstrate their capacity to effectively induce apoptosis, a crucial process for eliminating cancer cells without the need for membrane permeabilization typical of conventional electroporation techniques. This ability to target intracellular structures, such as mitochondria, while having a limited impact on the plasma membrane represents a significant advantage for the therapeutic use of nsPEFs [31]. However, several limitations persist. Although nsPEFs are promising, they still require further engineering development and clinical research, particularly for treating internal tumors. This includes the development of specific electrode devices, such as catheter electrodes, to enable effective clinical application, especially during laparoscopic procedures. Moreover, while preclinical results are encouraging, further studies are needed to fully understand the mechanisms of action of nsPEFs and to confirm their long-term clinical efficacy. The complexity of the relationships between nsPEFs parameters (pulse duration, electric field intensity, and energy density) and the induction of apoptosis also complicates the prediction of therapeutic outcomes [32]. Recent studies have shown that applying nsPEFs at high frequencies (5 kHz) can maintain antitumor efficacy comparable to that achieved at lower frequencies while reducing unpleasant sensations such as the painful muscle contractions often associated with electroporation. Additionally, the use of these high frequencies can induce apoptosis, as observed through transmission electron microscopy (TEM), both in vitro and in vivo. However, it is essential to continue exploring the effects of these frequencies on the biomechanical properties of skeletal muscles and on the pain perception threshold for optimal clinical application [33]. In terms of risks, while nsPEFs are designed to minimize damage to healthy tissues, they do present potential adverse effects. For instance, electric fields could affect blood vessels, although the effects on large vessels seem to be limited. Additionally, it is crucial to understand intracellular mechanisms to avoid any undesirable effects, such as necrosis, especially when shorter pulse durations are used. It is crucial to recognize that an excessively strong electric field may cause unintended harm to surrounding tissues, highlighting the importance of optimizing treatment parameters [34]. Nanosecond pulsed electric fields offer a promising approach to cancer treatment, combining significant benefits with important technological and clinical challenges. Continued research is essential to develop this technology and ensure its safe and effective clinical application (Table 1).

### 2.2. Nanobubble-Assisted Ultrasound Cancer Therapy

Ultrasound, a form of mechanical energy, can be precisely focused to target tumor tissues, thereby minimizing collateral damage to surrounding healthy tissues. Traditional clinical use of ultrasound in cancer treatment primarily involves hyperthermia ablation using high-intensity focused ultrasound. The employment of micro- or nano-sized bubbles not only enhances imaging and improve diagnosis but also provides therapeutic effects with precise destruction of tumor tissues. Low-intensity ultrasound enhances therapeutic responses without significant thermal effects, making it more tolerable and more suitable for precision targeting. In addition, it requires relatively inexpensive equipment. Generally, there are two ways to apply nanobubble-assisted ultrasound for cancer treatment: one is sonodynamic therapy, and the other is targeted drug delivery. In this review, we discuss SDT only [44].

Sonodynamic therapy is similar to photodynamic therapy, but it utilizes ultrasound rather than light. A nanoparticle renders the target cell hypersensitive to ultrasound, leading to therapeutic effects. The term sonodynamic therapy also derives from photodynamic therapy. However, unlike photodynamic therapy, in which photosensitizers are excited directly by light to produce reactive oxygen species, sonodynamic therapy is mediated via ultrasound-induced cavitation and sonosensitizers to produce free radicals that kill nearby rapidly dividing cancer cells [45,46].

In sonodynamic therapy, the sonication parameters (usually 1.0–2.0 MHz at an intensity of 0.5 to 3.0 W cm^−2^) are selected to produce inertial cavitation in a cell culture or tumor, where micro- or nanobubbles rapidly collapse, resulting in shockwaves that produce free radicals and a cascade of molecular events that activate the sonosensitizer and, in turn, damage the cancer cells mechanically and chemically. Besides shock waves, processes such as sonoporation (which enhances cell membrane permeability), liposome disruption, and localized hyperthermia can also play a role in tumor tissue disruption [47].

Both cell study and animal model studies have proven that SDT holds great potential for targeted cancer treatment. For example, Perera et al. [48] demonstrated that SDT induced a significant increase in apoptosis (78.4 ± 9.3%, *p* < 0.01) of tumor cells in a mouse model, extending the median survival rate by 103% through tumor growth inhibition.

The advantages of nanobubble- and ultrasound-mediated therapies over traditional cancer treatments emphasize the non-invasive nature, precision targeting, and reduced systemic side effects. An attraction of sonodynamic therapy is its non-invasive treatment of deep body tumors. In contrast, photodynamic therapy uses visible light, which attenuates rapidly in tissues, has limited penetration, and can be employed only superficially or intra-operatively. Since nanobubble-assisted ultrasound also doubles as a tool for targeted drug delivery and imaging, SDT is oftentimes used in combination with other cancer diagnostic and therapies to develop innovative cancer theragnostic approaches. Future research needs to address the difficulties in controlling the penetration depth and intensity of ultrasound, as limited penetration highlights the limitations in treating certain tumor types, particularly those deeply located or surrounded by complex anatomical structures, where ultrasound penetration might be less effective. Potential side effects and safety concerns, including off-target effects, tissue damage from cavitation, and nanobubble toxicity, require further investigation (Table 2) [49].

### 2.3. Photoablation for Tumor Reduction

**Figure 3:**
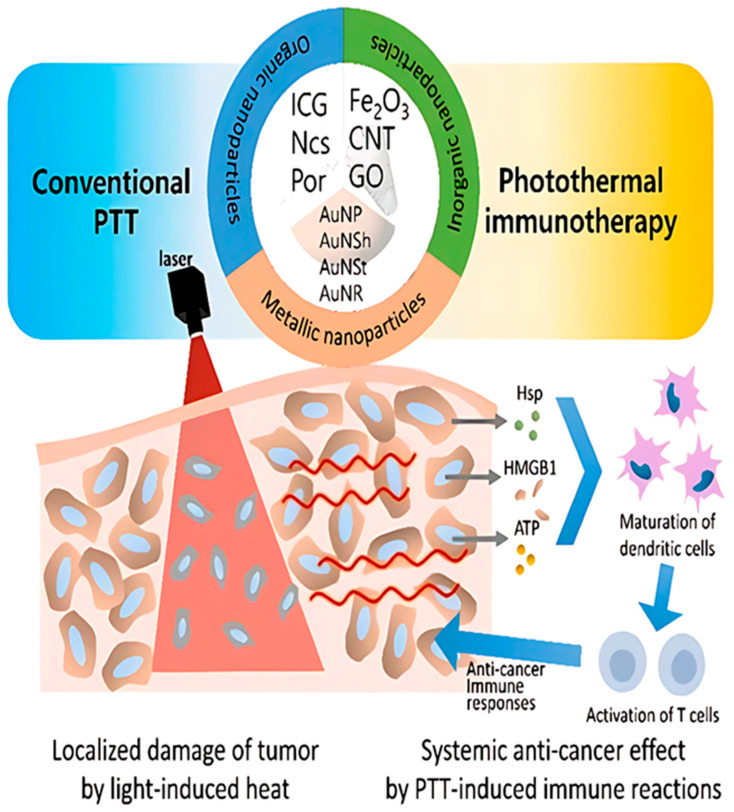
Diagrammatic representation of photothermal treatment (PTT) nanoparticles based on therapeutic principles. The targeted cancer’s cell death (apoptosis or necrosis) in the radiated area is prioritized in conventional PTT. Photothermal immunotherapy, on the other hand, targets the immune responses following PTT in order to provide a systemic anti-cancer impact. (The following are abbreviations: GO (graphene oxide); AuNP (gold nanoparticles); AuNSh (gold nanoshells); AuNSt (gold nanostars); AuNR (gold nanorods); Hsp (heat shock protein); HMGB1 (high-mobility group box 1); ATP (adenosine triphosphate); PTT (photothermal therapy); ICG (indocyanine green); Ncs (naphthalocyanines); Por (porphyrin); CNT (carbon nanotubes) [60].

Photoablation is a type of therapy that includes both photodynamic therapy (PDT) and photothermal therapy (PTT). Photothermal therapy (PTT) relies on heat generation to physically ablate cancer cells, whereas photodynamic therapy (PDT) utilizes the production of reactive oxygen species (ROS) to exert cytotoxicity [59]. Ablative therapy can also be delivered in a minimally invasive manner targeting deep lying organs and at the same time allowing less pain and a shorter recovery time. Often, nanoparticles help in increasing the effectiveness and accuracy of the treatment (Figure 3).

Additionally, nanoparticles provide a significant improvement in imaging-guided afterglow luminescence. Photothermal therapy (PTT), among other ways, has gained significance in intercellular biological studies thanks to the great potential of gadolinium (Gd)- and dysprosium (Dy)-doped composites as single-doping components for theragnostic approaches based on upconverting nanoparticles. These nanoparticles can efficiently absorb near-infrared (NIR) laser light to transduce into heat for more efficacious tumoricidal treatment, and their magnetic property allows an imaging guidance for monitoring in the course of therapy. These days, photoablation and photothermal techniques are being increasingly utilized for the treatment of cancer in order to target tumor cells with high precision while also reducing damage to the surrounding healthy tissues [61]. Laser–tissue interaction can be described by three phenomena: scattering, reflection, and absorption. The light absorbed by tissue is converted into heat. Prolonged exposure of tumor cells at temperatures ranging from 45 °C to 55 °C or short exposure at temperature higher than 60 °C causes irreversible cell damage, thus causing tumor reduction. Heat generation in the tissue, hence the effect of LA, is influenced by a number of factors: laser light wavelength, laser settings (laser power, laser energy, and treatment time), physical properties of the tissue, and emission characteristics of the optical applicator [62].

Hepatocellular carcinoma (HCC) and liver metastases are the most commonly treated cancers by Nd:YAG lasers. These treatments are performed with low power measured in Watts and the time of treatment measured in minutes (e.g., 5 W and 6–12 min). Laser power may be increased to 30 W–40 W. Premalignant lesions and early stages of penile cancer carcinoma have been treated with a combination of Nd:YAG and CO_2_ lasers. Bladder cancer was treated by Nd:YAG lasers with high power and short-duration pulses with laser power less than 35 W to avoid the risk of perforating the bowel or bladder. After the 1990s, the Ho:YAG laser operating in pulsed mode at a wavelength of 2100 nm replaced the Nd:YAG laser for the treatment of superficial bladder cancer. This laser has been also used for the ablation of cervical lymph node metastases from papillary thyroid carcinoma with good results in terms of technical success [63]. Table 3 summarizes the laser parameters used for the treatment of various cancer types.

**Figure 4:**
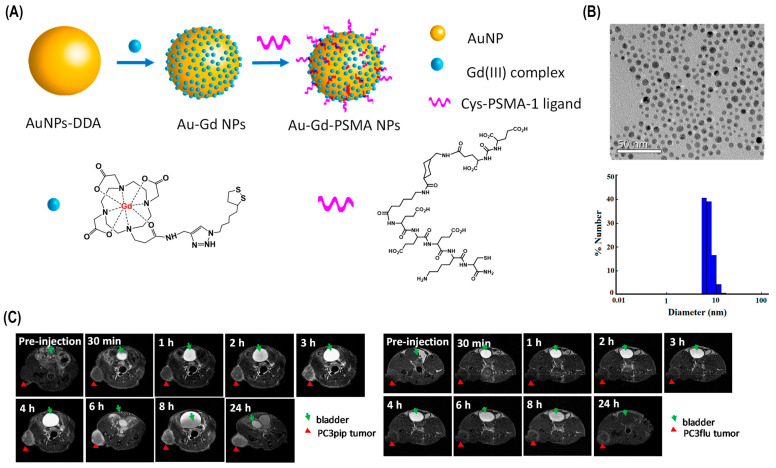
Au-Gd3+-PSMA NPs for MR-guided radiation therapy. (**A**) A schematic of Au-Gd3+-. PSMA NPs. (**B**) TEM micrograph demonstrates that Au-Gd3+-PSMA NPs have an average core size of 5 nm, while DLS shows a hydrodynamic diameter of 7.8 nm. (**C**) In vivo tumor targeting of Au-Gd3+-PSMA NPs and MR imaging of PC3pip and PC3flu mice at 7 T. PC3pip tumor cells display high PSMA levels, while PC3flu tumor cells express low PSMA levels [80].

Various other techniques in combination with photoablation have been used in cancer therapy for improved results. For instance, photoablation and more recent diagnostic imaging modalities like CT and MRI have been employed in combination for more precise and targeted tumor elimination. MR thermometry and CT thermometry were established as non-invasive techniques of tumor reduction. Another successful technique is the synergetic strategy of forming red blood cell-based gels, and PTT is able to simultaneously ablate tumors and activate immune responses, resulting in more accurate tumor-specific rejection (Figure 4).

But the most noteworthy solution has been the use of nanoparticles in the photothermal ablation of cancer. The aim of this solution is to improve the selectivity of the treatment in order to destroy the tumor while preserving the integrity of the healthy surrounding tissue. The basis of this therapy is that materials that highly absorb light can be designed and delivered specifically to the tumor cells. The subsequent application of light will then cause the specific thermal killing of nanoparticle-tagged tumor cells. Moreover, employing biocompatible nanoparticles, e.g., indocyanine green (ICG), in current laser ablation techniques elevates accumulation at tumor sites, promoting prolonged therapeutic effects and leading to reduced recurrence rates [41]. The former use of multifunctional hybrid reduced graphene oxide nanoparticles (rGO NPs) has been found important in cancer therapy, especially in photothermal therapy (PTT), a recent area of nanomedicine. These nanoparticles offer a unique platform for integrating pH-tunable diagnostic capabilities alongside targeted therapeutic interventions, enhancing the precision and efficacy of treatment. A significant advancement in this field involves decorating rGO NPs with a PEG-g-PDMA-quaternized IR825 and HA anchoring system, enabling precise control over fluorescence quenching properties. This construct improves the photothermal aspect by building a fine-tuned photocage-based heat-generating system in response to exogenous illuminations such as near infrared (NIR) irradiation, which leads more significant therapeutic outcomes [81].

The size, shape, and chemical structure of gold nanoparticles dictates the optical properties due to the interaction of light with the free electrons of the gold surface, a phenomenon referred to as localized surface plasmon resonance (LSPR) [82]. There have been a variety of different types of gold nanoparticles explored in the NIR region, including gold/silica nanoshells, nanorods, and nanocages. The biocompatibility of gold and the ability to conjugate biologically relevant molecules to its surface through the sulfur–gold interaction, including polyethylene glycol (PEG) for stealth capabilities and antibodies for targeting, make it ideal as a therapeutic and diagnostic tool [83]. Bare gold nanoparticles are utilized for in vitro applications, whereas PEG-coated gold nanoparticles are employed for in vivo applications. In vitro testing shows that gold/gold sulfide nanoparticles in combination with nIR laser light can cause photothermal destruction of tumor cells. In vivo testing provided bio-distribution data showing the gold/gold sulfide nanoparticles can remain in circulation longer than gold/silica nanoshells, for longer than 24 h based on neutron activation analysis (NAA) and longer based on dynamic light scattering (DLS). Survival data show an effective photothermal therapy, with survival greater than 80% for optimized accumulation times. With further optimization of laser power parameters and nanoparticle concentrations, gold/gold sulfide nanoparticles could provide an alternate therapeutic option that could prove very effective, combined with gold/silica nanoshells, which are currently being evaluated clinically; these particles could complement the treatment options for particular types of cancers [84]. Cell-staining assays demonstrated the high biocompatibility of these nanoparticles and their cancer cell selectivity uptake in vitro as well as photothermal lysis against cancer cells. Additional ex vivo biodistribution studies have bolstered the specificity of these agents, confirming the most significant uptake into neoplastic tumor cells [85].

The current landscape of LA is changing rapidly, with new and exciting developments [86]. Among others, emerging solutions and developments that are noteworthy include the recent evolution in the use of new lasers with different wavelengths and modes of operation and equipment (e.g., custom applicators), leading to promising results in terms of treatment selectivity, and the improved understanding of the laser–tissue interactions. Interestingly, clinical translations of photoablation on different diseases beyond cancer have also been reported and are used to correct specific disorders, including corneal diseases, where photoablation improves visual acuity and reduces astigmatism. Moreover, advanced studies involving brain-mimicking phantoms are being used to refine laser–tissue interaction models, providing insights into optimizing photoablation therapies for brain tumors [86] (Table 4).

## 3. Nascent Inorganic Nanoparticles for Drug-Free Cancer Therapy

### 3.1. Yttrium-90 Microsphere Radioembolization for Cancer Therapy2.1. Apoptosis Induction with Nanosecond Pulsed Electric Fields

**Figure 5:**
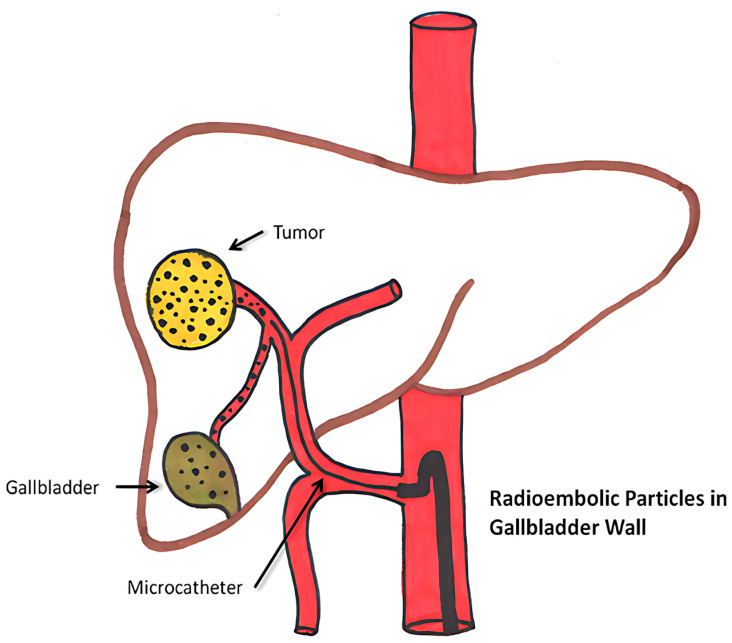
Diagrammatic depiction of abnormal microsphere accumulation on the gallbladder wall [98].

Yttrium 90 radioembolization is a minimally invasive, catheter-based procedure. The technique involving yttrium-90 is based on the principles of embolization, which entails the occlusion of blood flow to a tumor, combined with radiotherapy, wherein radioactive microspheres are administered directly to the tumor site. To deliver microspheres, usually, a small incision is made in the artery of the groin or wrist region using a catheter, which is a thin, flexible tube [97]. It is primarily used for treating liver cancers, which cannot be removed surgically. This treatment is particularly effective for hepatocellular carcinoma (HCC), the most common type of liver cancer, as well as for intrahepatic cholangiocarcinoma (ICC) and liver metastases from cancers like colorectal, breast, and neuroendocrine tumors (Figure 5).

The half-life of Y-90 is around 64.2 h, and it penetrates 2.5–11 mm inside the tissue. It emits pure beta radiation. They are irreversibly integrated into glass or resin microspheres, and the size variation observed in glass is between 20–30 μm and in resin ranges from 20 to 60 μm. It enables 90 Y microspheres to deliver up to 94% of the radiation dosage during the first 11 days of treatment before the radiation decays into stable zirconium. With a mean energy of 0.94 MeV and a maximum power of 2.26 MeV, β-radiation is released by yttrium-90 microspheres trapped in the liver’s microvasculature. The two types of yttrium-90 microspheres that are commercially available are TheraSphereTM (BTG, London, UK) and SIR-Spheres™ (Sirtex Medical Limited, NSW, Australia), which have microspheres made of glass and resin, respectively [99].

Yttrium-90 radioembolization therapy can cause hepatic, extrahepatic, and systemic complications. Hepatic side effects include hepatic fibrosis, portal hypertension, and bilirubin toxicity. Rare effects like GI ulcers, radiation pneumonitis, and dermatitis may occur due to microsphere misplacement or radiation exposure, which can be categorized as extrahepatic complications. Systemic impacts include lymphopenia, thrombocytopenia, and rare vascular injuries or pancreatitis. Furthermore, microsphere accumulation in or close to the gallbladder may result in cholecystitis, which is uncommon. These side effects highlight the need for precise procedural methods and close patient monitoring [100].

Recent advancements in this field aim to mitigate side effects through several innovative approaches. Building on these advancements, the novel sol–gel method for yttrium-90 microsphere production further refines treatment precision. This approach minimizes off-target radiation and enhances durability by creating uniform, chemically stable microspheres with high specific activity (190 MBq/mg). These improvements ensure more accurate trans-arterial radioembolization, significantly reducing side effects and improving patient outcomes in liver cancer therapy [101]. Other developments in imaging for yttrium-90 radioembolization include advanced PET/CT systems utilizing positron emitters for superior resolution and quantitative accuracy in dosimetry. Artificial intelligence (AI) and machine learning algorithms are now being integrated to analyze imaging data, predict outcomes, and optimize treatment plans. These innovations complement traditional SPECT advancements, providing even greater precision in targeting liver tumors and safeguarding healthy tissues [102].

Another technique that can assist in validation is MIM SurePlan software for post-treatment PET-based dosimetry in Y-90 radioembolization, which enables precise dose mapping. By ensuring accurate radiation dose calculations, it minimizes exposure to healthy tissues, reducing side effects and enhancing the safety and efficacy of liver cancer treatments [103] (Table 5).

### 3.2. Epigenetic Reprogramming Using Engineered Nanoparticles

**Figure 6:**
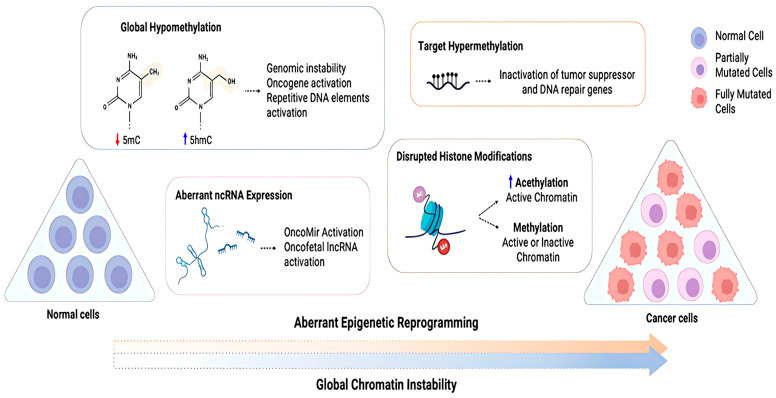
Diagram depicting cancer cells’ abnormal epigenetic reprogramming. Cancer cells exhibit global chromatin instability due to aberrant reprograming of normal cells, which includes loss of global methylation, altered ncRNA expression, disrupted histone modifications, and hypermethylation of target genes. This abnormal reprograming can inactivate tumor suppressor and DNA repair genes while activating oncogenes. 5-methylcytosine (5mC) and 5-hydroxymethylcytosine (5hmC) are defined [110].

Epigenetic reprogramming plays a crucial role in normal development of cells and is vital for preserving the unique epigenetic patterns specific to different cell types during cell division. These epigenetic modifications are, however, very dynamic and reversible, and as a result, an individual’s genetic makeup might strongly be influenced by the environmental conditions they are living in and their lifestyle [109]. Due to this instability, any disruptions that might be caused in the natural epigenetic program of gene expression might trigger the development of tumors, leading to cancer. Several different types of epigenetic modifications are thought to contribute to tumorigenesis, including those that alter chromatin structure, modify DNA and histones, remodel nucleosomes, and incorporate variant histones (Figure 6).

Engineered nanoparticles (ENPs) on the other hand, either artificial or natural, can deliver agents, genes, or proteins for therapeutic and/or diagnostic purposes, entering cells via different routes and with the capacity to be functionalized for specific therapeutic purposes. An ENP has novel/different physical properties, such as an increased surface area to volume ratio, reactive sites, charge, shape, mobility, and thermal properties. While travelling between cells, certain specifically designed ENPs can either directly or indirectly interact with lipids, proteins, and nucleic acids, causing multiple changes at the molecular or cellular level [111,112].

Among the intentionally engineered NPs, metal oxide NPs are the most widely produced and used nanomaterials for epigenetic reprogramming. It has been demonstrated that exposure to metals often may lead to toxicity, altered gene expression, changes in epigenetic marks, and metal-induced carcinogenesis. Thus, combining the characteristic benefits of ENPs and the importance of regulating epigenetic changes, a novel approach to bring about epigenetic changes by introducing ENPs into the cell body has been popularized [113,114].

Epigenetic changes can be brought about by three major reprogramming techniques: DNA methylation, histone modifications, and non-coding RNA interactions. These can alter chromatin structure and DNA accessibility by establishing a differential gene expression program in a cell specific manner, without bringing changes to the DNA sequence 73. This article reviews various nanoparticles specifically designed for the reprogramming techniques mentioned above, which can alter the epigenetic structure of cancer tumors and provide therapeutic benefits. So far, research has been conducted using both animal and human cell lines. Clinical trials, however, have been very limited [115] (Table 6).

#### 3.2.1. DNA Methylation

DNA methylation, being the most common epigenetic change, has been studied extensively using various types of engineered nanoparticles, and the effects have been tested both in animal and human cell lines. Some of the most extensively studied nanoparticles are gold (AuNPs), silver (AgNPs), and other metal oxide nanoparticles like titanium oxide (TiO_2_), zinc oxide (ZnO), and silicon oxide or silica (SiO_2_) nanoparticles; single- and multi-walled carbon nanotubes (SWCNTs and MWCNTs); graphene quantum dots; etc. Metal oxide nanoparticles are the most commonly generated and utilized nanomaterials among the purposefully designed NPs [119,120]. A 2015 study on pregnant mice found that AgNPs (silver nanoparticles) alter DNA methylation, reducing methylation levels in the Zac1 and Igf2r DNA regions in treated placentas (animal model 1). Among various nanoparticles, AuNPs (gold nanoparticles) were found to be more potent than CNTs (carbon nanotubes) in inducing epigenetic changes. Another study using lung fibroblast (MRC5) cells showed that sublethal doses of TiO_2_ and ZnO nanoparticles induced epigenetic changes, with TiO_2_ being more toxic than ZnO [119]. Additionally, research on HaCaT cells exposed to SiO_2_ nanoparticles demonstrated increased DNA methylation of the PARP-1 promoter, decreased global DNA methylation, and reduced levels of methyltransferases (Dnmt1, Dnmt3A, and MBD2). These findings highlight the potential of various nanoparticles to induce epigenetic modifications [121]. Further, it was discovered that size also played an important role in determining the therapeutic effect brought about by the nanoparticles. Gold nanoparticles of three primary sizes (5 nm, 60 nm, and 250 nm) and both single- and multi-walled CNTs were tested in the BALB/c mice cell line (animal model 2), revealing that more genes were epigenetically affected by AuNP 60 nm than 5 or 250 nm. Further, when compared to the control, the total cell count was higher with exposure to CNTs; however, it was concluded that CNT exposure had no effect on 5-methylcytosine (5mc) and 5-hydroxy methyl cytosine (5hmc) in mouse lungs [122,123]. Interestingly, SWCNTs and MWCNTs also had shape effects on promoter methylation of ATM (ataxia-telangiectasia mutated (ATM) protein), and ATM genes normally help prevent cancers. A mutation in this gene causes a disruption in its regular functions. This could imply that exposure to these nanotubes might influence how the ATM gene is regulated at the epigenetic level, potentially impacting its expression and, consequently, cellular responses to DNA damage [124].

Other important factors that determine the therapeutic effects of these nanoparticles on cancer cells are incubation time and concentration. In MRC5 cells treated with TiO_2_ NPs, DNA methylation was reduced to 49% after 24 h and 48% after 48 h. For ZnO-exposed cells, DNA methylation decreased to 43% and 42% at 24 and 48 h, respectively. At concentrations of 0.5 and 1 μg/mL, DNA methyltransferase activity was significantly repressed by about 50% for TiO_2_ and 40% for ZnO within 24 h. This suppression persisted at higher concentrations (4 μg/mL and 8 μg/mL) but was unaffected by longer incubation times. The study revealed that NPs reduced genomic DNA methylation in a concentration-dependent manner, with no significant impact from extended exposure. These findings suggest that NPs induce DNA hypomethylation as early as 24 h, with no additional reduction over time, highlighting their concentration-dependent effects on DNA methylation and cellular responses [125] (Table 7).

#### 3.2.2. Histone Modifications

Apart from changes in DNA methylation brought about by nanomaterials and nanoparticles, another epigenetic reaction is an alteration of typical histone modification patterns. Similar to DNA methylation, histone modifications are also a dynamic process that is influenced by the balance between histone phosphorylases, acetyltransferases, and methyltransferases that are responsible for introducing a particular chemical histone modification and histone phosphatases, deacetylases, and demethylases that are responsible for removing chemical histone modifications. Several studies have been conducted to demonstrate the substantial disruption of histone modification patterns as a result of exposure to nanomaterials and nanoparticles [134].

Histone modifications happen in the accessible N-terminal region. Histone acetyltransferases (HATs) stimulate hyperacetylation of the histones, causing activation of genes while histone deacetylases (HDACs) stimulate deacetylation, thereby causing chromatin condensation and gene silencing and development of cancer. HDAC inhibitors stimulate cancer cell arrest, apoptosis, and differentiation, thereby reactivating normal cell processes. With chemotherapeutic drugs and these inhibitors, there seems to be hope for breast cancer treatment. Mutations in the methylating enzyme activity were found in other cancers such as bladder, melanoma, and lung. An understanding of epigenetic reversion has enabled the discovery of drugs to target HDACs that can be used to forestall tumor growth [135].

Nanoparticles play an important role in inducing histone modifications, too.

One of the most consistent alterations induced by exposure to a broad range of nanomaterials and nanoparticles is increased phosphorylation of histone H2AX at serine-139 (ɣ-H2AX). When tested on certain cell lines, such as lung adenocarcinoma epithelial cells A549, MDA-MB-231, and MDA-MsB-468 breast cancer cells and human skin keratinocytes (HaCaT), etc., TiO_2_, SiO_2_, ZnO, CuO, silver, and gold nanoparticles have increased ɣ-H2AX. When the mechanism of histone modification alterations induced by nanoparticles was researched, it was found that one of the most common effects of NMs and NPs is the induction of cellular stress, e.g., oxidative and endoplasmic reticulum stress, and metabolic disturbances, e.g., one-carbon metabolism and the citric acid cycle. These events might in turn lead to DNA damage, low repair response, and metabolic alterations affecting the functioning of chromatin-modifying enzymes [136].

A recent study suggested that the architecture of nanoparticles plays an important role in reprogramming the epigenome, specifically in histone modifications. When the effect of AuNPs was tested on the A549 cell line, it was found that the star-shaped “spiky” AuNPs were the ones inducing demethylation of di- and tri-methylation lysine 27 (H3K27me2/3) and not the spherical ones. This revelation may be explored more to guide the development of nano-based therapies to target aberrant epigenetic patterns associated with cancer [137].

#### 3.2.3. Non-Coding RNA Expression

Non-coding RNAs (ncRNAs) play crucial roles in cancer development through epigenetic regulation. Long non-coding RNAs (lncRNAs) and microRNAs (miRNAs) influence gene expression by interacting with epigenetic modifiers and transcription factors 86. The subcellular localization of lncRNAs is important for their regulatory functions in cancer progression. N6-Methyladenosine (m6A) modification in both coding and non-coding RNAs affects cancer pathogenesis and drug response [138].

Nanoparticle-based delivery systems have been developed to regulate lncRNA expression for cancer therapy by overcoming barriers to nucleic acid delivery in vitro and in vivo. These systems efficiently transport nucleic acids, such as siRNA and DNA, to silence overexpressed lncRNAs or deliver downregulated ones, improving therapeutic impact while reducing toxicity. Common nanoparticles include lipid-based, polymeric, inorganic, and bio-inspired NPs. A recent example of lipid-based NPs is the mRNA-1273 developed by Moderna therapeutics for coronavirus disease in 2019. It facilitated the usage of lipid NPs to deliver synthetic mRNA encoding the spike protein of the coronavirus into human cells. Once inside the cells, the mRNA is translated into the spike protein, prompting the immune system to produce antibodies and T-cell responses to recognize and fight the virus if exposed in the future [139,140]. Inspired by their success, lipid-based NPs are widely used for siRNA delivery in cancer therapy, such as Lock et al.’s nanoplatform for acute myeloid leukemia treatment. Polymeric NPs offer advantages like ease of functionalization, tunable properties, and low cost. The polymeric NPs are constructed by modifying both natural (e.g., chitosan and cellulose) and synthetic polymers (e.g., poly(lactide-co-glycolide) and poly (lactic acid)) with low-molecular-weight polyethyleneimine and amino-containing compounds. This enables the polymers to directly complex nucleic acids by compromising for the lack of a cationic moiety in their structures [141].

Inorganic NPs, such as AuNPs, offer flexibility in size, shape, and real-time tracking through imaging techniques. For example, AuNPs enhanced antisense oligonucleotide delivery, silencing MALAT1 in lung cancer cells. Biomimetic NPs have been recently emerged as a new class of drug carriers that are capable of mimicking the biological features and functions of native cells. One type of representative biomimetic NPs are cell-derived exosomes, which mimic biological functions and offer low immunogenicity, targeting cells naturally. A cell study conducted by Zheng and coworkers showed that exosomes loaded with lncPTENP1 suppressed bladder cancer proliferation and migration in studies. At present, further research to develop methods that help improve the accurate targeted delivery of biomimetic NPs for altering lncRNA expression and using it for cancer therapy is still ongoing [142].

In conclusion, epigenetic reprogramming through engineered nanoparticles offers a promising approach for cancer therapy, particularly by targeting mechanisms such as DNA methylation, histone modifications, and non-coding RNA interactions. The ability of nanoparticles to alter the epigenetic landscape highlights their therapeutic potential, as demonstrated by studies on various ENPs, including metal oxide nanoparticles, carbon nanotubes, and biomimetic exosomes. Still, there are challenges in optimizing nanoparticle design for particular subcellular targets, such as the endoplasmic reticulum and ribosomes, and improving the accuracy and efficiency of epigenetic modulation. While promising progress has been made in preclinical studies, clinical trials for nanoparticle-based epigenetic therapies are scarce [143]. Future research should be focused on the exploration of novel epigenetic markers for DNA methylation and histone modifications, ensuring the long-term stability of epigenetic changes, analyzing the complex functions of lncRNAs with respect to their subcellular localization, and refining the nanoparticle delivery system to increase therapeutic outcomes while reducing toxicity. These areas will hold the key to unlocking the full potential of ENP-mediated epigenetic reprogramming for cancer treatment [144] (Table 8).

## 4. Targeting the Tumor Microenvironment, Precision Medicine, and Immunotherapy for Personalized Cancer Treatment

### 4.1. Tumor Microenvironment: A Key Focus in Cancer Therapy

**Figure 7:**
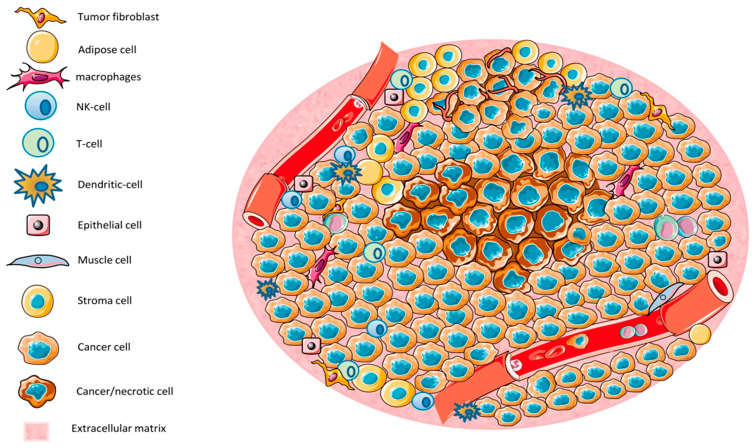
The tumor microenvironment (TME) consists of various cell types, including tumor cells, immune cells, epithelial cells, and stromal cells. Regions characterized by low nutrient and oxygen levels lead to necrosis [154].

The tumor microenvironment (TME) is a complex and dynamic system composed of tumor cells, immune cells, stromal cells, extracellular matrix components, and signaling molecules. It plays a crucial role in cancer progression, metastasis, and resistance to therapies (Figure 7). Various immune cells within the TME, such as tumor-associated macrophages (TAMs), myeloid-derived suppressor cells (MDSCs), T cells, natural killer (NK) cells, dendritic cells (DCs), and regulatory T cells (Tregs), significantly influence tumor development and therapeutic responses. Immune cells in the TME exhibit distinct phenotypes and functions, often promoting tumor immune evasion and immunosuppression [152,153].

These immune cells are involved in a variety of mechanisms, including the release of cytokines, chemokines, and growth factors that shape the immune landscape of the tumor. TAMs, for instance, are abundant in most cancers and exhibit a pro-tumorigenic phenotype, promoting tumor growth and metastasis through the secretion of cytokines and matrix metalloproteinases (MMPs). TAMs can polarize into two distinct subsets: M1 and M2. M1 macrophages have anti-tumorigenic properties, while M2 macrophages are immunosuppressive and contribute to tumor progression. In addition to macrophages, MDSCs are another major population of immunosuppressive cells in the TME [155]. MDSCs consist of two main subsets: monocytic MDSCs (M-MDSCs) and granulocytic MDSCs (G-MDSCs). These cells suppress anti-tumor immunity by producing immunosuppressive molecules like arginase-1, reactive oxygen species (ROS), and nitric oxide (NO). T cells also play a critical role in the TME, and their functions can be altered by the immunosuppressive nature of the microenvironment. Cytotoxic CD8+ T cells are essential for anti-tumor immunity, but in many cancers, they are rendered dysfunctional or exhausted due to prolonged exposure to tumor-derived signals, such as programmed cell death protein 1 (PD-1) ligands and immune checkpoint molecules [156]. This phenomenon, known as T-cell exhaustion, leads to a reduced ability of T cells to mount effective anti-tumor responses. NK cells, which are also part of the innate immune system, are crucial in tumor surveillance. However, their activity is often impaired in the TME due to the presence of inhibitory cytokines and immune checkpoint molecules. Dendritic cells (DCs), on the other hand, are important for initiating immune responses by presenting tumor antigens to naïve T cells [157]. However, in the TME, DCs often undergo functional suppression due to the presence of immunosuppressive factors, limiting their ability to prime an effective anti-tumor immune response. Regulatory T cells (Tregs) are another critical population within the TME. These cells suppress immune responses and promote tolerance to tumor cells. They can inhibit the activity of cytotoxic T cells and NK cells, thereby allowing tumors to evade immune surveillance. The presence of Tregs in the TME is associated with poor prognosis in many cancers, as their accumulation promotes immunosuppression. The extracellular matrix (ECM) within the TME is composed of various proteins, including collagen, fibronectin, and laminin, which provide structural support and regulate tumor cell behavior [158]. The ECM also serves as a scaffold for immune cells and facilitates their interactions with tumor cells. Dysregulation of the ECM can promote tumor progression and metastasis by enhancing tumor cell invasion and migration. The TME also contains various soluble factors, including cytokines, growth factors, and exosomes, which regulate immune cell function and contribute to immune suppression. For example, transforming growth factor beta (TGF-β) is a potent immunosuppressive cytokine found in many cancers [159]. TGF-β suppresses the activity of cytotoxic T cells and NK cells, promotes the differentiation of Tregs, and enhances the immunosuppressive functions of TAMs and MDSCs. Other factors, such as interleukins (IL-10 and IL-6), vascular endothelial growth factor (VEGF), and indoleamine 2,3-dioxygenase (IDO), also contribute to the immune-suppressive environment of the TME. The complex interactions between immune cells, stromal cells, and the ECM in the TME create a unique immunosuppressive microenvironment that hinders the effectiveness of immune-based therapies [160]. The manipulation of the TME, either by targeting immune cells or modifying the ECM, holds promise for improving cancer immunotherapy outcomes. Recent advances in immuno-oncology have led to the development of immune checkpoint inhibitors, which aim to block the inhibitory signals in the TME and restore the anti-tumor immune response. Drugs like pembrolizumab and nivolumab, which target PD-1, and ipilimumab, which targets cytotoxic T-lymphocyte-associated protein 4 (CTLA-4), have shown promise in treating various cancers by reinvigorating T-cell responses. Moreover, therapies aimed at reprogramming TAMs, depleting Tregs, or targeting MDSCs are also being investigated to enhance anti-tumor immunity. Understanding the intricate relationships between the immune cells, ECM, and soluble factors within the TME is critical for developing more effective and personalized cancer therapies. These advances represent exciting opportunities for overcoming immune evasion and improving the clinical outcomes of cancer patients [161].

### 4.2. Precision Medicine: Tailored Solutions for Better Outcomes

**Figure 8:**
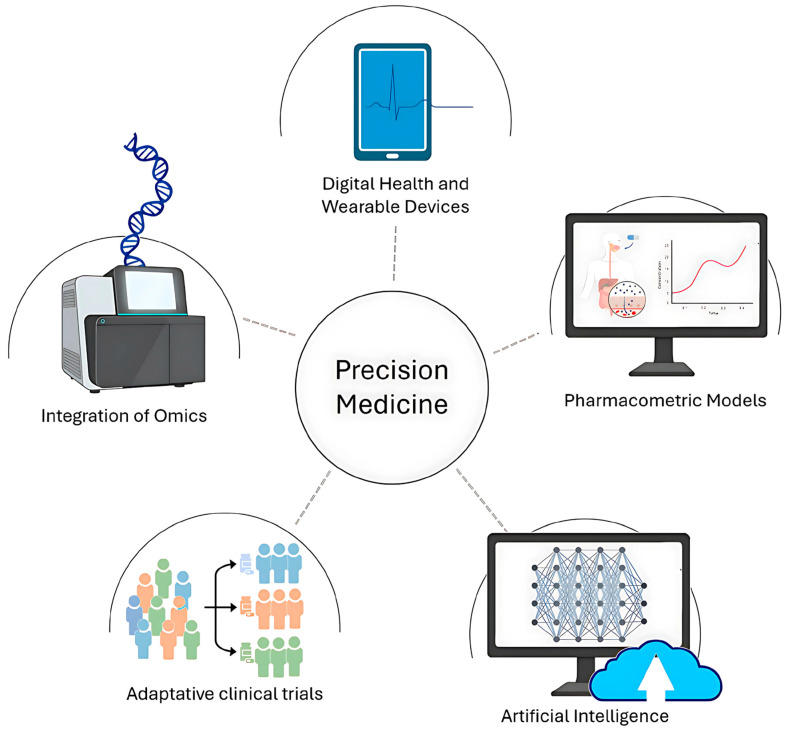
Schematic illustration of precision medicine [162].

Precision medicine represents a paradigm shift in healthcare by moving away from generalized treatments and instead delivering tailored interventions based on an individual’s unique genetic, molecular, and environmental factors. This approach aims to improve clinical outcomes by targeting the underlying biological mechanisms of diseases in a patient-specific manner. However, achieving the goals of precision medicine requires significant advancements in identifying genetic variants, analyzing multi-omics data, and understanding their clinical implications. The success of precision medicine relies heavily on genomics, particularly the identification of specific gene variants or mutations correlated with clinical outcomes [162,163,164,165] (Figure 8).

This process is complex, requiring extensive research to uncover genetic polymorphisms associated with disease phenotypes or therapeutic responses. It begins with DNA sequencing, a technology that has evolved significantly since the completion of the Human Genome Project (HGP) in 2003. The HGP was a 13-year endeavor led by the International Human Genome Sequencing Consortium (IHGSC), involving over 200 laboratories in 19 countries. The project revealed that humans have approximately 20,500 genes and share 99.99% of their genomic DNA, with only 0.01% of the genome contributing to individual genetic differences (International Human Genome Sequencing Consortium, 2004) [166]. Within this small percentage lie single nucleotide polymorphisms (SNPs) and other variations, many of which have potential as disease biomarkers or predictors of therapeutic outcomes. Early genome sequencing relied on bacterial artificial chromosomes (BAC) and Sanger sequencing. BACs were instrumental in mapping DNA fragments to specific chromosomal locations, while Sanger sequencing provided precise base-by-base identification of these fragments. Despite their accuracy, these methods were costly and time-consuming. For example, the HGP cost an estimated USD 3 billion to complete. Today, next-generation sequencing (NGS) technologies have replaced these early techniques, offering 1000-fold reductions in cost and enabling sequencing of entire genomes for as little as USD 1000 [167]. NGS technologies revolutionized genomic research by allowing high-throughput, parallel sequencing of DNA fragments. These methods enable scientists to sequence an individual’s genome in a matter of days, providing an unprecedented ability to identify genetic variants, including rare mutations. Despite their efficiency, interpreting the vast datasets generated by NGS remains a major challenge. For instance, correlating genetic variations with disease predisposition or treatment outcomes often requires bioinformatics tools capable of handling terabytes of data [168].

Additionally, genomics alone does not fully capture the complexity of human biology. Studies have shown that genetic factors explain only 30–50% of disease variability, with environmental, epigenetic, and other molecular factors accounting for the rest. To bridge the gap between genotype and phenotype, precision medicine integrates other “omics” technologies, including transcriptomics, proteomics, and metabolomics [169,170]. Each layer provides insights into distinct biological processes, creating a comprehensive view of a patient’s health. Transcriptomics focuses on the complete set of mRNA transcripts in a cell or tissue, known as the transcriptome. This field provides crucial insights into how genes are expressed and regulated. Two primary techniques dominate transcriptomic research: microarray analysis and RNA sequencing (RNA-Seq). Microarray technology measures mRNA expression by hybridizing sample RNA with complementary probes. The intensity of fluorescence indicates the expression levels of specific genes. While microarrays are cost-effective and have standardized protocols, they require prior knowledge of gene sequences, limiting their ability to detect novel transcripts [171]. RNA-Seq offers a more advanced approach, capable of identifying both known and novel transcripts without requiring pre-designed probes. By sequencing mRNA directly, RNA-Seq provides greater sensitivity, a lower signal-to-noise ratio, and the ability to analyze smaller sample sizes (e.g., nanograms vs. micrograms for microarrays). However, RNA-Seq is more expensive, though its costs are expected to decline as protocols become more standardized. Transcriptomics has already transformed drug development by linking gene expression patterns to disease phenotypes and therapeutic effects. For example, transcriptomic profiling can identify patients likely to respond to immunotherapy, such as those with high expression of PD-L1 in cancer. Proteomics analyzes the complete set of proteins in a cell or organism, including their structure, abundance, and interactions. Since proteins are the functional molecules mediating biological processes, proteomics is critical for understanding disease mechanisms and identifying therapeutic targets [172].

The most widely used technique in proteomics is mass spectrometry (MS), which can quantify protein levels, detect post-translational modifications, and characterize protein-protein interactions. Two primary MS strategies are employed: bottom-up proteomics and top-down proteomics [173]. Bottom-up proteomics (shotgun proteomics) breaks down proteins into smaller peptides for analysis, enabling the identification of complex mixtures. However, this approach can lose information about specific proteins and is biased toward high-abundance proteins. Top-down proteomics analyzes intact proteins, preserving information about modifications and structure but requiring advanced instrumentation. Recent advancements in MS labeling techniques allow for simultaneous analysis of multiple samples, making proteomics more efficient. For instance, proteomic studies have identified biomarkers like HER2 in breast cancer, which guides the use of trastuzumab (Herceptin) therapy [174,175,176]. Metabolomics examines the small molecules (metabolites) involved in cellular metabolism, providing real-time insights into physiological states. Metabolites reflect both genetic and environmental influences, making metabolomics a powerful tool for understanding complex diseases. For example, metabolic profiling has identified unique signatures associated with diabetes, cardiovascular diseases, and cancer. While the integration of multi-omics data holds immense promise, several challenges remain [177]. These include data interpretation, as the vast and complex datasets generated by omics technologies require advanced bioinformatics tools; cost and accessibility, as advanced omics technologies remain inaccessible in many regions; and ethical concerns, such as the use of genetic data in relation to privacy and potential misuse by insurers or employers [178]. Despite these hurdles, the future of precision medicine looks promising. Emerging technologies, such as single-cell sequencing and multi-omics integration platforms, are expected to further personalize treatments. Additionally, collaborative efforts like the All of Us Research Program aim to sequence the genomes of one million individuals to advance our understanding of individual variability. Precision medicine represents a transformative approach to healthcare, leveraging genomic, transcriptomic, proteomic, and metabolomic data to deliver tailored treatments. Advances in next-generation sequencing, mass spectrometry, and bioinformatics are paving the way for more personalized and effective therapies. While challenges remain, ongoing research and technological innovation continue to push the boundaries of what precision medicine can achieve. As these tools become more accessible and integrated, precision medicine will play a central role in improving patient outcomes worldwide [152,179].

### 4.3. Immunotherapy: Empowering the Immune System to Fight Cancer

Immunotherapy, especially immune checkpoint inhibitors, has transformed cancer treatment by harnessing the body’s immune system to identify and eradicate malignant cells. James Allison and Tasuko Honjo’s significant contributions to the understanding of CTLA-4 and PD-1 pathways facilitated the development of FDA-approved monoclonal antibodies, including ipilimumab, nivolumab, and pembrolizumab [180]. These inhibitors operate by obstructing immune checkpoints that typically inhibit T-cell activity, thereby reinstating the immune system’s capacity to identify and eliminate tumor cells. Immune checkpoint inhibitors have markedly enhanced survival rates in patients with advanced cancers, including melanoma, non-small cell lung cancer (NSCLC), renal cell carcinoma, and Hodgkin lymphoma. Despite their success, not all patients exhibit effective responses to checkpoint inhibitors, prompting continued research into combination therapies and biomarker-driven strategies to improve response rates [181]. The capacity of the immune system to identify cancer cells depends on antigen presentation via major histocompatibility complex (MHC) molecules. Dendritic cells are essential for presenting tumor-specific antigens to naive T cells within the lymph nodes. The interaction mediated by the T-cell receptor (TCR) necessitates supplementary co-stimulatory signals, chiefly the binding of CD28 on T cells to B7.1/CD80 and B7.2/CD86 on antigen-presenting cells. Tumors utilize immune checkpoints such as CTLA-4 and PD-1 to avoid detection by the immune system [182]. CTLA-4 competes with CD28 for B7 ligands, thereby inhibiting early T-cell activation, whereas PD-1 binds to its ligands PD-L1 and PD-L2, leading to the suppression of effector T-cell function in the tumor microenvironment [14,183]. The blockade of inhibitory receptors through monoclonal antibodies reinstates immune surveillance and enhances sustained antitumor responses. Ipilimumab, a checkpoint inhibitor targeting CTLA-4, was the first to show clinical efficacy in metastatic melanoma. Preclinical studies in mice demonstrated that anti-CTLA-4 therapy increased CD8+ T-cell activation and facilitated tumor rejection. Clinical trials subsequently confirmed that ipilimumab enhanced overall survival in patients with advanced melanoma. Its use is linked to immune-related adverse events (irAEs), such as colitis, dermatitis, and endocrinopathies, resulting from excessive immune activation [184]. The identification of PD-1 by Honjo’s team offered a novel therapeutic target characterized by an improved safety profile. Nivolumab and pembrolizumab, both PD-1 inhibitors, have shown significant efficacy in melanoma, non-small cell lung cancer (NSCLC), and renal carcinoma, yielding durable responses and enhanced progression-free survival relative to chemotherapy [185]. These drugs operate by inhibiting PD-1-mediated T-cell exhaustion, thus preserving a strong immune response against tumors. The efficacy of immune checkpoint inhibitors exhibits considerable variability among patients, with response rates ranging from 20% to 40% in specific cancers. This variability has prompted extensive investigation into predictive biomarkers, including tumor mutational burden (TMB) and PD-L1 expression. A high tumor mutational burden (TMB) is associated with enhanced neoantigen presentation, thereby increasing tumor vulnerability to immune response. Cancers exhibiting elevated PD-L1 expression, including smoking-related NSCLC, demonstrate a greater likelihood of responding to anti-PD-1 therapy. The KEYNOTE-024 trial indicated that pembrolizumab markedly enhanced overall survival in NSCLC patients exhibiting PD-L1 expression greater than 50%, resulting in its endorsement as a first-line therapy [186,187]. Resistance mechanisms, such as the loss of antigen presentation and the upregulation of alternative immune checkpoints like TIM-3 and LAG-3, continue to pose significant challenges. Combination therapies that target these pathways are presently being studied. Researchers are investigating synergistic strategies that combine immunotherapy with conventional treatments such as chemotherapy and radiation to address primary and acquired resistance to checkpoint inhibitors [188]. Chemotherapeutic agents like oxaliplatin and cyclophosphamide induce immunogenic cell death, resulting in the release of tumor antigens that activate T cells [189,190]. Radiation therapy similarly enhances tumor antigenicity and promotes the activation of dendritic cells, thereby augmenting the effects of checkpoint blockade. The PACIFIC trial showed that the combination of durvalumab, a PD-L1 inhibitor, with chemoradiotherapy enhances survival in stage III NSCLC, thereby establishing a new standard of care. Emerging strategies utilizing T-cell agonists, including CD137 (4-1BB) and OX40, seek to enhance antitumor immunity by improving T-cell persistence and effector function. Promising developments encompass novel checkpoint targets like TIGIT and VISTA, which modulate immune responses through distinct mechanisms and are currently under investigation in clinical trials. Recent advancements in immunotherapy have concentrated on personalized cancer vaccines and adoptive cell therapies. Neoantigen vaccines, which are based on individual tumor mutations, elicit T-cell responses targeting distinct cancer antigens [189]. Clinical trials assessing mRNA-based vaccines alongside checkpoint inhibitors have demonstrated encouraging outcomes in melanoma and colorectal cancer. Chimeric antigen receptor (CAR) T-cell therapy, which has demonstrated efficacy in hematologic malignancies, is now being modified for application in solid tumors via innovative engineering approaches. Research is currently focused on CAR T cells that target mesothelin in pancreatic cancer and HER2 in glioblastoma. Bispecific T-cell engagers (BiTEs) link T cells to tumor cells through CD3 and tumor-associated antigens, providing an innovative method for enhancing immune-mediated cytotoxicity [191]. The advancement of next-generation immune-modulating agents, including STING agonists, TLR agonists, and oncolytic viruses, demonstrates potential in enhancing the effectiveness of immunotherapy. CRISPR-based gene editing is utilized to improve T-cell function and mitigate exhaustion, facilitating the development of more effective cellular therapies. While immune checkpoint inhibitors have achieved notable success, issues of accessibility and affordability persist, especially among low-income populations [192]. The substantial expense of monoclonal antibody therapies, frequently surpassing USD 100,000 per patient each year, restricts their broad implementation. Initiatives from governments and pharmaceutical companies to offer immunotherapy as a free or subsidized treatment have increased, focusing on expanding access. The development of biosimilars and alternative manufacturing strategies, including cell-free protein synthesis, presents potential solutions for reducing production costs. Global health organizations advocate for the inclusion of immune checkpoint inhibitors in essential medicine lists to promote equitable distribution. Current clinical trials are examining cost-effective small molecule inhibitors that regulate immune checkpoints, which may offer a more economical option compared to antibody-based therapies [193,194]. The advancement of immunotherapy depends on optimizing treatment approaches to enhance effectiveness and reduce adverse effects. Current research is investigating the combination of immune checkpoint inhibitors with innovative agents aimed at the tumor microenvironment, including IDO1 inhibitors, adenosine receptor antagonists, and therapies for macrophage reprogramming. Advancements in single-cell sequencing and spatial transcriptomics are improving our comprehension of tumor-immune interactions, facilitating the development of next-generation immunotherapies. The discoveries of Allison and Honjo have initiated a significant shift in oncology, converting previously untreatable cancers into manageable conditions. Ongoing research is elucidating novel mechanisms of immune evasion, thereby making the objective of rendering immunotherapy a viable and curative option for all cancer patients more achievable [195].

## 5. Traditional Medicine

Traditional medicine has long been regarded as a holistic, drug-free approach to managing various health conditions, including cancer, by addressing the interconnectedness of physical, emotional, and spiritual well-being. Rooted in ancient systems such as Traditional Chinese Medicine (TCM), Ayurveda, and Indigenous healing practices, these modalities emphasize restoring balance within the body and enhancing innate healing capacities. Unlike conventional pharmacological treatments, traditional therapies often focus on lifestyle modifications, manual techniques, and mind–body interventions to mitigate symptoms, improve quality of life, and support recovery. For instance, TCM’s concept of Qi (vital energy) and Ayurveda’s emphasis on balancing doshas (biological energies) underpin many practices aimed at reducing tumor progression, alleviating treatment side effects, and fostering resilience [196]. The World Health Organization (WHO) recognizes traditional medicine as a valuable component of healthcare, particularly in low-resource settings, and advocates for its integration into modern oncology when supported by evidence (WHO, 2019). While these approaches are rarely curative on their own, their role in palliative and supportive care has gained traction, especially as patients increasingly seek complementary strategies to augment conventional therapies like chemotherapy and radiation [197].

One of the most studied traditional modalities in cancer care is acupuncture, a cornerstone of TCM that involves stimulating specific points on the body with fine needles to regulate Qi flow. Research suggests acupuncture may modulate neurological and immune responses, offering relief from chemotherapy-induced nausea, chronic pain, and cancer-related fatigue. A study by Garcia et al. [198]. found that breast cancer patients undergoing acupuncture reported significant reductions in pain and improved energy levels compared to control groups. The National Cancer Institute (NCI) acknowledges its potential in managing side effects such as neuropathy and xerostomia, though it underscores the need for standardized protocols to ensure consistency in clinical outcomes. Beyond symptom management, preclinical studies explore acupuncture’s role in inhibiting tumor angiogenesis and enhancing the efficacy of anticancer drugs, though these findings remain preliminary. Skeptics caution against overinterpretation of small-scale studies, emphasizing the placebo effect and variability in practitioner skill. Nevertheless, the growing inclusion of acupuncture in oncology guidelines reflects its acceptance as a low-risk adjunct therapy [199].

In addition to physical interventions, mind–body practices such as meditation, yoga, and qigong are integral to traditional systems and have been widely adopted in cancer care. These practices aim to harmonize mental and physical states, reducing stress and enhancing emotional resilience, which are critical for patients navigating the psychological toll of cancer. For example, mindfulness-based stress reduction (MBSR) programs, rooted in Buddhist meditation traditions, have demonstrated measurable benefits in reducing anxiety, depression, and sleep disturbances among cancer survivors [200,201]. Carlson et al. [202]. reported that participants in an MBSR program experienced lower cortisol levels and improved immune function, suggesting a biological basis for these psychological benefits. Similarly, yoga, which combines postures, breathwork, and meditation, has been shown to alleviate fatigue and improve functional mobility in patients undergoing chemotherapy. A Cochrane review by Cramer et al. [203] concluded that yoga significantly enhances quality of life and reduces treatment-related distress, though it noted heterogeneity in study designs and outcomes. These practices are particularly appealing for their accessibility and adaptability, allowing patients to tailor them to their physical limitations or cultural preferences. Movement-based therapies such as tai chi and qigong, which originated in TCM, offer another dimension of drug-free cancer support by integrating gentle physical activity with meditative focus. These practices emphasize fluid movements, deep breathing, and mental concentration to enhance energy flow and physical stamina. A randomized controlled trial by Mustian et al. [204] involving breast cancer survivors found that tai chi not only reduced inflammation and fatigue but also improved cognitive function and cardiovascular health, with effects comparable to those of conventional aerobic exercise. Qigong, often described as “moving meditation”, has shown promise in mitigating cancer-related fatigue and improving emotional well-being, particularly in patients with advanced disease. Such therapies are especially valuable for individuals who find rigorous exercise challenging due to treatment-related debilitation. While the mechanisms underlying their benefits such as modulation of inflammatory cytokines or vagal nerve activation are still under investigation, their safety profile and holistic impact make them a pragmatic addition to integrative oncology programs.

Dietary and herbal interventions derived from traditional systems also play a significant role in non-pharmacological cancer care. Ayurveda, for instance, prescribes personalized nutrition plans, detoxification rituals (Panchakarma), and herbal formulations like turmeric (*Curcuma longa*) and ashwagandha (*Withania somnifera*) to strengthen the body’s defenses and reduce oxidative stress. Curcumin, the active compound in turmeric, has been extensively studied for its anti-inflammatory and antiproliferative properties, with preclinical evidence suggesting it may sensitize cancer cells to chemotherapy. Similarly, Traditional Chinese dietary therapy advocates for foods with “cooling” or “warming” properties to counteract imbalances linked to cancer progression. The Mediterranean diet, inspired by traditional eating patterns, is another example lauded for its high content of antioxidants and omega-3 fatty acids, which may suppress chronic inflammation and tumor growth. However, the clinical application of these dietary approaches requires careful customization, as overly restrictive diets or unregulated herbal use may interfere with conventional treatments or exacerbate malnutrition. Collaboration between oncologists and traditional practitioners is essential to navigate these complexities [205].

Despite their potential, traditional therapies are not without challenges. Critics highlight the lack of large-scale, rigorous clinical trials, inconsistent regulatory standards, and the risk of medicine interactions with conventional treatments. For example, certain herbs may inhibit or potentiate the metabolism of chemotherapy drugs, leading to toxicity or reduced efficacy. The Society for Integrative Oncology (SIO) stresses the importance of evidence-based integration, urging patients to disclose all traditional therapies to their healthcare team to avoid adverse interactions [206]. Moreover, cultural appropriation and commercialization of traditional knowledge raise ethical concerns, particularly when practices are divorced from their cultural context or monetized without benefiting originating communities. Ensuring equitable access and respecting the intellectual property of Indigenous healers remain pressing issues in global health discourse. Looking ahead, the future of traditional medicine in oncology lies in bridging ancient wisdom with modern science. Advances in epigenetics, metabolomics, and psychoneuroimmunology are beginning to unravel the biological mechanisms underlying practices like meditation and acupuncture, lending credibility to their therapeutic claims. For instance, research on the gut–brain axis has illuminated how mindfulness practices may alter microbiome composition, influencing immune responses to cancer. Similarly, studies on electroacupuncture have mapped its effects on neural pathways that regulate pain and inflammation. Multidisciplinary collaborations such as the WHO’s Global Center for Traditional Medicine aim to foster research, standardize training, and develop policies that ensure safe, equitable integration. As cancer care increasingly shifts toward personalized and patient-centered models, traditional drug-free therapies are poised to play a pivotal role in comprehensive survivorship programs, offering tools to empower patients and address the multidimensional challenges of cancer [207].

## 6. Clinical Translation

The effective clinical translation of drug-free cancer therapies requires the thorough research, validation, and establishment of standardized protocols to guarantee efficacy and safety. A key challenge is the translation of laboratory findings into practical applications. Preclinical studies have shown promising results; however, the heterogeneity of cancer types and patient responses necessitates extensive clinical trials to confirm reproducibility and generalizability. Researchers must refine experimental models, enhance the scalability of treatment modalities, and develop reliable biomarkers for patient selection to address this issue. Regulatory approval represents a critical challenge in the implementation of drug-free therapies in clinical settings. Conventional cancer therapies adhere to established regulatory processes, while novel drug-free approaches necessitate the development of new assessment frameworks. Regulatory bodies like the FDA and EMA should develop new guidelines that take into account the distinct mechanisms of action associated with energy-based therapies and nanoparticle-driven interventions. Compliance with ethical standards, execution of multi-phase trials, and demonstration of long-term safety and efficacy are essential for achieving regulatory acceptance and integration into mainstream oncology. In addition to regulatory approval, the prioritization of patient safety is essential. Numerous drug-free therapies, including high-intensity focused ultrasound and nanosecond pulsed electric fields, function based on principles that are markedly distinct from traditional chemotherapy or radiotherapy. The challenge involves optimizing treatment parameters to reduce off-target effects and enhance tumor selectivity. Long-term studies evaluating the effects of these interventions on the immune system, organ function, and potential secondary malignancies are essential to confirm their efficacy as primary or adjunctive cancer treatments. The integration of drug-free cancer therapies with existing treatment regimens is a critical factor in their clinical translation. These therapies should not be considered independent solutions; instead, they must serve as complementary strategies that can augment the efficacy of conventional treatments. Combining photothermal therapy with immunotherapy may enhance the immune response to tumors, thereby improving long-term outcomes. Similarly, integrating nanotechnology-based approaches with precision medicine allows for targeted delivery of therapeutic agents, minimizing toxicity and improving patient tolerance. A further challenge pertains to the cost and accessibility of these therapies. Advanced nanomaterials and specialized energy-based devices necessitate substantial investment in their manufacturing and distribution processes. Addressing the financial burden on healthcare systems and patients requires policy reforms, enhanced insurance coverage, and the implementation of cost-effective production strategies. Ensuring equitable access to these treatments across various socioeconomic backgrounds is essential for their widespread adoption. Government funding, collaborations between academia and industry, and partnerships between the public and private sectors are essential for enhancing affordability and accessibility. Technological advancements, including artificial intelligence and machine learning, present significant opportunities for enhancing the clinical translation of drug-free therapies. Algorithms powered by artificial intelligence can aid in treatment planning, forecast patient responses, and enhance therapy parameters in real-time. Integrating computational models with experimental and clinical data enables researchers to expedite the development and refinement of personalized cancer treatment strategies. The ongoing advancement of diagnostic imaging, robotic-assisted delivery systems, and real-time monitoring tools will enhance the precision and efficiency of drug-free interventions.

## 7. Conclusions

The evolution of cancer treatment has led the scientific community to a critical juncture in the quest for safer, more effective, and personalized therapeutic approaches. Conventional cancer treatments significantly enhance patient survival; however, they are associated with notable limitations such as severe side effects, systemic toxicity, and the emergence of resistance. Drug-free cancer therapies offer a novel strategy, utilizing sophisticated physical, biological, and technological methods to selectively target cancer cells. These therapies include various techniques such as high-intensity focused energy beams, nanosecond pulsed electric fields, photothermal therapy, and the use of inorganic nanoparticles to induce tumor apoptosis without pharmacological agents. These methods provide a novel approach to oncological treatment by reducing collateral damage to healthy tissues. The integration of drug-free therapies with precision medicine and immunotherapy represents a significant advancement, enabling the development of more personalized treatment regimens. Modifying the tumor microenvironment, activating the immune system, and targeting cancer at a molecular level present significant opportunities for enhancing patient outcomes. Utilizing nanoparticles as carriers for energy-based interventions facilitates targeted delivery while minimizing off-target effects, thereby decreasing systemic complications commonly linked to traditional therapies. Techniques like photothermal therapy, which employs near-infrared-responsive nanoparticles, enable precise ablation of tumor tissues while safeguarding surrounding healthy structures, thereby enhancing control over the treatment process.

Despite significant advancements, considerable challenges persist that must be addressed before drug-free cancer therapies can achieve mainstream acceptance. Challenges including treatment scalability, reproducibility, long-term safety, and regulatory approval represent significant obstacles that necessitate comprehensive research and clinical validation. Ensuring biocompatibility and stability of nanomaterials, optimizing energy parameters for focused interventions, and refining imaging technologies for real-time monitoring are critical steps for the clinical viability of these therapies. Additionally, it is essential to develop strong regulatory frameworks that address the complexities associated with nanotechnology and non-drug-based interventions to facilitate their clinical adoption. The accessibility and affordability of drug-free cancer therapies are crucial for their success. The integration of these techniques into mainstream healthcare systems will depend on their cost-effectiveness and practical implementation. Advancements in material science, bioengineering, and computational modeling are anticipated to significantly enhance these methods, increasing their affordability and accessibility. Interdisciplinary collaborations among researchers, clinicians, industry stakeholders, and policymakers are essential for translating laboratory findings into effective clinical solutions that benefit a broader patient population. The future of drug-free cancer therapy appears promising, as ongoing research advances the frontiers of innovation. The integration of artificial intelligence, machine learning, and real-time imaging technologies can improve the precision and effectiveness of these treatments. Utilizing big data analytics and predictive modelling enables clinicians to customize interventions for individual patients, thereby enhancing therapeutic outcomes while minimizing risk. The ongoing enhancement of non-invasive and minimally invasive techniques is expected to establish a new standard of care in oncology, characterized by safer, more effective treatment regimens tailored to the individual biological profiles of patients.

In conclusion, drug-free cancer therapies signify a significant advancement in oncological treatment, providing safer and more targeted alternatives to conventional methods. Leveraging advancements in physics, nanotechnology, bioengineering, and immunotherapy, these methods have the potential to transform cancer treatment. Despite the presence of substantial challenges, ongoing research and interdisciplinary collaboration are essential for addressing these issues and establishing drug-free therapies as a standard option in clinical oncology. With advancements in science, the objective of converting cancer into a manageable and potentially curable condition while minimizing systemic toxicity is becoming more achievable. The future of cancer treatment is rooted in innovation, with drug-free therapies exemplifying the ongoing quest for safer and more effective solutions in combating cancer.

## Figures and Tables

**Table 1:** Comparison of nsPEFs, electroporation, pharmacological treatments, and thermal ablation, focusing on mechanisms, selectivity, and clinical benefits.

Aspect	Nanosecond Pulsed Electric Fields (nsPEFs)	Conventional Electroporation	Pharmacological Cancer Treatments	Thermal Ablation Techniques	References
Mechanism	High-intensity nanosecond pulses (10–300 ns) induce apoptosis without membrane damage.	Creates pores via longer pulses (micro- to milliseconds) for drug/gene delivery.	Cytotoxic drugs disrupt DNA synthesis, cell division, or signaling.	Heat (lasers, radiofrequency) destroys tumors via protein denaturation.	[35]
Energy Source	High-frequency electric pulses via specialized electrodes.	Electric pulses for drug/gene delivery.	Systemic/local chemotherapeutic agents.	Lasers, radiofrequency, or microwaves.	[36,37]
Tissue Penetration	Deep and localized; targets internal tumors (e.g., liver, melanoma).	Limited penetration (depends on pulse parameters).	Systemic (widespread but toxic).	Superficial/moderate depth; risks overheating healthy tissues.	[38,39]
Selectivity	High (targets cancer cells via dielectric properties and larger nuclei).	Low (damages nearby cells).	Moderate (depends on drug targeting).	Low (collateral tissue damage).	[40]
Pain/Discomfort	Minimal pain at high frequencies (5 kHz); mild muscle contractions at low frequencies.	Low-moderate discomfort (depends on pulse frequency/placement).	Severe side effects (nausea, immunosuppression).	Pain from thermal spread; requires anesthesia.	[41]
Cost and Accessibility	Moderate upfront costs; potential long-term cost effectiveness.	Moderate (device-dependent).	High long-term costs (drug resistance, toxicities).	Variable (energy-intensive equipment).	[41]
Recurrence Rates	Low recurrence (angiogenesis/metastasis suppression).	Moderate (requires precise calibration).	High (drug resistance common).	Moderate (risk of incomplete ablation).	[42,43]

**Table 2:** Comparison of SDT, PDT, traditional ultrasound, and nanobubble-assisted ultrasound therapies, highlighting mechanisms, precision, safety, and clinical applications.

Aspect	Sonodynamic Therapy (SDT)	Photodynamic Therapy (PDT)	Traditional Ultrasound Therapy	Nanobubble-Assisted Ultrasound	References
Mechanism	Ultrasound + sonosensitizers → free radicals via cavitation.	Light + photosensitizers → ROS generation.	High-intensity ultrasound → thermal ablation.	Nanobubble cavitation enhances imaging/therapeutic targeting.	[50,51]
Energy Source	Ultrasound (1.0–2.0 MHz, 0.5–3.0 W/cm^2^).	Visible/NIR light.	Ultrasound (HIFU/LIFU).	Ultrasound + nanobubbles.	[52]
Treatment Depth	Deep penetration (suitable for liver, pancreas).	Shallow (~1–2 cm).	Deep tissues.	Deep tissues (enhanced by nanobubble cavitation).	[53]
Targeting Precision	High (tunable ultrasound parameters + sonosensitizers).	Moderate (depends on light focus + photosensitizer targeting).	Moderate (thermal spread risks).	High (nanobubbles enable selective cavitation).	[54]
Energy Efficiency	High (localized free radicals minimize energy waste).	Moderate (light attenuation reduces efficiency).	Moderate (energy-intensive hyperthermia).	High (cavitation amplifies therapeutic effects).	[55]
Safety Profile	Non-invasive; cavitation may damage tissue at high intensity.	Safe for superficial use; photosensitizer toxicity possible.	Thermal damage to healthy tissues.	Safe with careful calibration; nanobubble stability critical.	[56]
Imaging Compatibility	Real-time monitoring (ultrasound imaging).	Limited (requires separate imaging).	Requires MRI/CT for monitoring.	Excellent (enhances imaging + therapy in one platform).	[57]
Theragnostic Potential	Combines therapy + real-time imaging.	Limited to treatment.	Imaging separate from therapy.	High (diagnostics + drug tracking).	[51,58]

**Table 3:** Comprehensive comparison of laser therapies across cancer types, highlighting mechanisms, benefits, limitations, side effects, and innovations.

Cancer Type	Laser Used	Laser Power (W) or Energy	Time (Minutes or Sessions)	Mechanism of Action	Clinical Benefits	Limitations	Emerging Innovations	Side Effects	Patient Eligibility	Treatment Costs	Efficacy Rate (%)	References
Hepatocellular carcinoma	Nd:YAG	30–40	6–12	Vaporizes tumor tissues through thermal ablation.	Effective for localized tumors; minimizes systemic effects.	Risk of thermal damage to surrounding tissues.	Integration with imaging guidance for precision targeting.	Pain, fever, localized edema.	Patients with localized HCC lesions, good liver function.	Moderate to high	~70–85%	[64]
Liver metastases	Nd:YAG	5	6–12	Localized hyperthermia causes tumor cell destruction.	Non-invasive approach with precise control over tumor ablation.	Limited to surface or accessible lesions; less effective for deep metastases.	Advanced catheter-based Nd:YAG systems for laparoscopic applications.	Pain, potential tissue damage.	Patients with isolated liver metastases, operable lesions.	Moderate to high	~65–80%	[65]
Premalignant lesions	Nd:YAG and CO2	N/A	N/A	Combined thermal and vaporization effects.	Useful for removing superficial lesions; reduces risk of progression.	Effective only for early-stage or surface lesions.	Synergistic use with photodynamic therapies for enhanced lesion targeting.	Redness, scarring, temporary swelling.	Patients with early-stage or superficial premalignant lesions.	Low to moderate	~85–95%	[66]
Bladder cancer	Nd:YAG	<35	Short time	Vaporizes tumor tissues; limits blood loss during ablation.	Effective for superficial bladder tumors; minimal bleeding.	Ineffective for deep or muscle-invasive tumors.	Combining laser therapy with immunotherapy to reduce recurrence rates.	Urinary retention, mild discomfort.	Patients with superficial bladder tumors, non-invasive candidates.	Moderate to high	~75–90%	[67]
Skin cancer (non-melanoma)	Solid-state, diode, dye lasers	200–500 J/cm^2^ (energy)	Varies	Ablates tumor tissues; selectively targets cancer cells.	High efficacy for basal cell carcinoma (BCC) and some SCC.	Lower efficacy for advanced or metastatic cancers.	Use of vascular-selective and ablative lasers for precision targeting.	Redness, mild pain, temporary pigmentation changes.	Patients with BCC, SCC, or early-stage localized lesions.	Low to moderate	~90–98%	[68]
Lung cancer	Argon dye laser	100–2000 mW	10–30	Uses hematoporphyrin derivative as a photosensitizer to enhance ROS.	Effective for early-stage lung cancer; reduces need for invasive surgery.	Limited by light penetration; effective only for centrally located tumors.	Composite-type optical fiberscope for accurate tumor irradiation.	Mild pain, coughing, light sensitivity.	Patients with early-stage, centrally located lung tumors.	High	~60–75%	[69]
Glioblastoma	Nd:YAG (LITT)	High (customized)	Varies	Focused thermal ablation of deep-seated gliomas.	Cytoreduction of glioblastomas in non-resectable regions.	Requires advanced imaging; risk of edema or brain damage.	Real-time MRI-guided LITT for monitoring ablation zones.	Brain swelling, edema, neurological deficits.	Patients with non-resectable glioblastomas, confirmed MRI lesions.	High	~50–70%	[70]
Head and neck cancer	Low-level laser (LLLT)	10–60 mW	Frequent sessions	Reduces inflammation and pain; promotes tissue healing.	Effective for preventing and treating oral mucositis during radiotherapy.	Limited to supportive care; does not treat the primary tumor.	Portable LLLT devices for home-based management of therapy side effects.	None reported for LLLT use in supportive care.	Patients undergoing radiotherapy, experiencing mucositis.	Low	N/A	[71]
Breast cancer	Indium gallium aluminum laser	660 nm (wavelength)	5 days/week during RT	Promotes tissue repair and prevents radiodermatitis.	Reduces pain and skin damage associated with radiation therapy.	Limited to radiation therapy side effects; does not address primary tumors.	Integration with advanced radiotherapy protocols for comprehensive care.	Skin redness, mild irritation.	Breast cancer patients undergoing radiotherapy.	Low	N/A	[72,73,74]
Prostate cancer	Nd:YAG (LITT)	High (customized)	Varies	Focal ablation of tumor via interstitial laser fiber.	Precise ablation with minimal impact on surrounding tissues.	Limited long-term data on recurrence and survival.	Multiparametric MRI for identifying treatment-related changes post LITT.	Mild urinary symptoms, tissue swelling.	Localized prostate tumors, low-grade cases.	High	~60–80%	[75]
Pancreatic cancer	Nd:YAG	20–40	10–15	Thermal ablation disrupts tumor cells in hard-to-reach areas.	Potential for treating unresectable tumors; non-invasive alternative.	Risk of thermal damage to vital structures; limited long-term data.	Laser-based combinatory therapies with immuno-oncology approaches.	Pancreatitis risk, localized pain.	Unresectable pancreatic cancer with no metastasis.	Moderate	~50–65%	[76]
Colorectal cancer	Nd:YAG or diode laser	10–20	5–10	Localized thermal necrosis of tumor tissues.	Effective in palliative care for tumor-related obstructions.	Only useful for palliative treatment; does not address systemic metastases.	Hybrid laser-catheter systems for minimally invasive interventions.	Mild rectal pain, bleeding.	Patients with advanced disease needing palliative care.	Low	~40–60%	[77,78]
Esophageal cancer	Argon plasma laser	1–10 W	Varies	Ablates surface tumors or clears obstructions.	Useful for palliative care in advanced stages; reduces dysphagia.	Ineffective as a curative option; risk of perforation or thermal injury.	Development of safer, automated systems for lesion ablation.	Esophageal irritation, localized pain.	Patients with advanced esophageal cancer needing palliation.	Low	~50–65%	[79]

**Table 4:** Comprehensive comparison of photothermal therapy (PTT) and photodynamic therapy (PDT), highlighting nanoparticle roles, laser parameters, energy efficiency, safety, and emerging applications.

Aspect	Description	(PTT)	(PDT)	Nanoparticle Types	Technical Specifications	Clinical Outcomes	Innovations & Future Directions	References
Mechanism	Conversion of absorbed light into heat for tumor cell destruction or ROS generation for cytotoxicity.	Heat generation physically ablates tumor cells by raising temperature above 45 °C.	Produces ROS for cytotoxic effects on tumor cells, inducing apoptosis or necrosis.	Gold NPs, rGO NPs, indocyanine green (ICG), Gd/Dy-doped NPs, PEG-coated NPs.	*Laser Parameters:* - Nd:YAG: 5–40 W for 6–12 min (HCC, liver metastases). - Ho:YAG: Pulsed mode at 2100 nm (bladder cancer).	*Clinical Benefits:* - Minimally invasive, preserves healthy tissue, enhances immune responses.	*Applications Beyond Cancer:* - Corneal disease correction, brain phantom optimization, astigmatism treatment.	[87,88,89]
Energy Source	Lasers (e.g., Nd:YAG, Ho:YAG, CO2) are used to provide localized light energy to initiate therapy.	NIR lasers (e.g., 800–1100 nm for deep penetration).	Visible light for shallow tumors; UV or NIR for deeper tissues.	Gold NPs (high biocompatibility), rGO NPs (pH-sensitive theragnostics), ICG (tumor-targeting agent).	*Energy Efficiency:* - Highly efficient due to localized energy deposition. - Low heat diffusion.	*Safety Profile:* - Reduced systemic toxicity with localized treatment. - High biocompatibility (e.g., PEGylated NPs).	*Emerging Technologies:* - pH-sensitive rGO NPs, multimodal imaging-guided ablation systems.	[90,91]
Thermal Effects	Heat generated directly by the absorbed light destroys tumor cells while sparing healthy tissues.	High temperature (45–55 °C prolonged exposure or >60 °C short exposure) kills tumor cells.	Not heat-based; works through ROS-mediated pathways causing oxidative damage to cancer cells.	Enhances selective thermal damage via NP tagging and improves imaging contrast.	*Treatment Depth:* - NIR penetrates deep tissues (up to several cm). - PDT limited to ~1–2 cm.	*Recurrence Rates:* - Low recurrence with NP targeting (e.g., rGO). - Higher recurrence in PDT if targeting fails.	*Future Applications:* - Cardiac fibrosis, bacterial biofilm removal, chronic disease drug delivery.	[92]
Theragnostics	Dual-purpose approach combines therapy (ablation) with diagnostics (imaging guidance, biodistribution).	Magnetic Gd/Dy-doped NPs allow imaging guidance for tumor localization and monitoring during PTT.	ROS production can be monitored with fluorescent or afterglow luminescence nanoparticles.	Gd/Dy NPs (theragnostics), PEG-coated AuNPs (enhanced circulation and targeting).	*Laser Parameters (Advanced):* - Wavelength/power vary by application (e.g., 2100 nm for Ho:YAG).	*Challenges:* - Optimize NP tumor specificity. - Improve ROS efficiency and NP stability.	*Innovative Therapies:* - Photoablation + immune checkpoint inhibitors for immunotherapy.	[93]
Nanoparticle Features	Biocompatibility, tumor selectivity, prolonged circulation, multifunctionality (e.g., imaging + therapy).	rGO NPs tuned for fluorescence quenching and heat generation, enhancing selectivity and efficacy.	Hybrid nanoparticles (e.g., PEG-coated gold NPs) improve ROS generation and reduce off-target effects.	NIR-responsive nanoparticles (e.g., gold nanorods, silica nanoshells) for deeper tissue penetration.	*Energy Efficiency (Optimized):* - Efficient heat conversion via NIR-responsive NPs.	*Safety (Advanced):* - Safe with calibrated laser intensity. - Biodegradable NPs reduce toxicity.	*Emerging Tools:* - Real-time MRI-thermometry for PTT. - Smart hydrogels for dual therapy.	[94]
Imaging Compatibility	Real-time guidance for improved precision during therapy; enables assessment of biodistribution and effectiveness.	Magnetic Gd/Dy NPs provide imaging capability via MRI guidance during PTT.	Luminescent imaging (fluorescent signals) tracks ROS generation and accumulation.	Gd/Dy NPs (MRI guidance), gold/silica nanoshells (optical tracking).	*Treatment Depth (Imaging-Guided):* - Deep penetration via imaging + NP delivery systems.	*Recurrence (Advanced):* - Real-time monitoring reduces residual tumor risk.	*Future Directions:* - Neurodegenerative/retinal disease treatments. - Precision targeting of infections.	[95,96]

**Table 5:** Comprehensive overview of yttrium-90 microsphere radioembolization, highlighting its applications, benefits, limitations, innovations, and clinical impact in cancer therapy.

Aspect	Details	Advantages	Disadvantages	Key Innovations	Imaging Techniques	Clinical Evidence	Applications Beyond Liver Cancer	Side Effects	Future Potential	Commercial Products	Patient Selection Criteria	Therapeutic Efficacy	References
Procedure Description	Minimally invasive catheter-based technique combining embolization and radiotherapy to target tumors directly.	Enables targeted delivery of radiation, minimizing damage to healthy tissues.	Requires advanced imaging and procedural expertise.	Sol–gel microsphere production for high-precision targeting.	PET/CT systems with improved resolution for accurate dosimetry.	Comprehensive reviews highlight significant efficacy in liver malignancies	Increasing use for pancreatic adenocarcinoma and metastatic breast cancer.	Hepatic fibrosis, portal hypertension, lymphopenia, and radiation pneumonitis.	Integration of AI and imaging to expand therapeutic indications and reduce toxicity.	*TheraSphere™:* Glass-based microspheres by BTG. *SIR-Spheres™:* Resin-based microspheres by Sirtex.	Patients with good liver reserve and low ECOG performance status benefit the most.	Median survival improved to 16.4 months in advanced HCC and colorectal metastases.	[104]
Primary Applications	Treats liver cancers such as HCC, intrahepatic cholangiocarcinoma (ICC), and liver metastases from colorectal, breast, and neuroendocrine tumors.	Effective for unresectable or chemotherapy-refractory cancers.	Limited to liver-specific malignancies in most current applications.	AI-driven treatment plans and advanced imaging for enhanced targeting of tumors.	SPECT combined with PET/CT for safer, precise delivery and post-therapy monitoring.	A phase III trial demonstrated improved time to progression (TTP) with radioembolization combined with fluorouracil compared to chemotherapy alone.	Expanding trials to metastatic pancreatic adenocarcinoma and other systemic cancers.	Cholecystitis and gastric ulcers are rare but possible.	Broadening indications through combination therapies and more durable microsphere designs.	Both products are widely approved for clinical use and demonstrate efficacy in liver-directed therapies.	Patients with unresectable malignancies but adequate hepatic function and manageable bilirubin levels.	Extended time to tumor progression demonstrated across multiple malignancies.	[105,106]
Mechanism	Combines tumor blood flow occlusion with delivery of β-radiation directly to the tumor.	Directs β-radiation with precision, delivering high-energy doses to cancer cells.	Risk of off-target radiation causing systemic side effects.	Personalized dosimetry using tools like MIM Sure Plan and advanced SPECT imaging.	Integration of 99mTc-MAA for pre-treatment planning and 90Y bremsstrahlung SPECT for post-treatment assessment.	Long-term studies highlight its utility for localized control in metastatic liver malignancies.	Potential to combine with immunotherapy for enhanced systemic tumor control.	Complication rates are low with proper imaging and microsphere placement.	Combining with systemic therapies like chemotherapy or immune checkpoint inhibitors for synergistic effects.	Advanced production ensures durable microsphere designs tailored for specific radiation delivery.	Selection based on performance status, tumor vascularity, and absence of significant extrahepatic disease.	Demonstrated efficacy in reducing tumor burden and improving survival in colorectal and HCC cases.	[107]
Radiation Properties	Half-life: ~64.2 h. Penetration: 2.5–11 mm. Mean energy: 0.94 MeV; Max energy: 2.26 MeV.	Short radiation half-life minimizes prolonged exposure to tissues.	Limited penetration range may reduce effectiveness in larger tumors.	High-specific-activity sol–gel microspheres enhance treatment precision and durability.	PET/CT imaging with positron emitters improves resolution and dosimetry accuracy.	European experience with Y-90 glass microspheres demonstrated median survival of 16.4 months in advanced HCC.	Exploring systemic benefits for metastatic colorectal and pancreatic tumors beyond the liver.	Radiation safety protocols minimize side effects; long-term risks are still under study.	Advanced microspheres with more consistent radiation activity to improve tumor control.	Increased demand for imaging-driven therapies supports global use in oncology centers.	Screening for prior chemotherapy failures and suitability for localized radiation therapy.	Survival benefits across multiple cancer types confirmed by meta-analyses.	[108]

**Table 6:** Comparative Analysis of Engineered Nanoparticles in Epigenetic Reprogramming.

Parameter	DNA Methylation	Histone Modifications	Non-Coding RNA Interactions
Mechanism of Action	Alters gene expression by adding/removing methyl groups (e.g., AgNPs reduce methylation in *Zac1* and *Igf2r*).	Modifies chromatin structure via acetylation, methylation, or phosphorylation (e.g., spiky AuNPs increase H2AX phosphorylation).	Delivers RNA therapies (siRNA/mRNA) to regulate lncRNAs/miRNAs (e.g., lipid NPs silence *MALAT1* in lung cancer).
Nanoparticles Used	AuNPs, AgNPs, TiO_2_, ZnO, SiO_2_, SWCNTs, graphene quantum dots.	TiO_2_, ZnO, SiO_2_, CuO, silver, gold nanoparticles (including “spiky” AuNPs).	Lipid-based, polymeric, inorganic NPs, biomimetic exosomes.
Experimental Models	Pregnant mice, BALB/c mice, MRC5 lung fibroblasts, HaCaT cells.	A549 lung adenocarcinoma cells, MDA-MB-231/468 breast cancer cells, HaCaT cells.	Lung cancer cells, acute myeloid leukemia cells, bladder cancer cell lines.
Key Findings	- AgNPs reduce placental DNA methylation. - Transient hypomethylation stabilizes quickly.	- Oxidative stress induces histone H2AX phosphorylation. - Spiky AuNPs enhance chromatin remodeling.	- Lipid NPs deliver siRNA to silence oncogenic lncRNAs (e.g., *MALAT1*). - Exosomes enable low-toxicity RNA delivery.
Factors Influencing Effects	Nanoparticle size, concentration, exposure time.	Nanoparticle architecture (e.g., “spiky” vs. spherical AuNPs).	Delivery system efficiency (e.g., lipid vs. polymeric NPs).
Therapeutic Applications	Reprogramming tumor suppressor genes (e.g., *Igf2r*) for cancer therapy.	Reactivating normal gene expression in tumors via histone acetylation/methylation.	Targeting dysregulated lncRNAs/miRNAs in cancers (e.g., leukemia, bladder cancer).
Challenges	- Ensuring long-term stability of epigenetic changes. - Minimizing nanoparticle toxicity.	- Refining NP design for specific histone targets. - Indirect oxidative stress effects.	- Subcellular RNA delivery barriers. - Enzymatic degradation of RNA.
**Future Directions**	- Developing novel epigenetic markers. - Optimizing NP size and concentration.	- Investigating NP-induced metabolic stress. - Refining “spiky” architectures for targeted chromatin remodeling.	- Enhancing biomimetic NPs (e.g., exosomes) for precision delivery. - Scalable production of RNA-loaded NPs.
**Advantages**	Targeted reprogramming of tumor suppressor/oncogene expression.	Reversible modifications allow dynamic control of gene activity.	Low toxicity (e.g., exosomes) and adaptability for RNA-based therapies.
**Limitations**	Early hypomethylation effects are transient; limited clinical trials.	Indirect effects of oxidative stress complicate therapeutic outcomes.	Delivery efficiency hindered by cellular uptake and enzymatic degradation.
**Clinical Trial Status**	Limited preclinical studies (animal models); no human trials yet.	Mostly in vitro and animal models; few human applications.	Emerging success (e.g., Moderna’s mRNA-1273 vaccine); translational potential.
**Commercial Viability**	Feasible for cancer diagnostics and therapies targeting epigenetic disruptions.	Requires NP design optimization for histone-specific applications.	Biomimetic NPs advancing but need R&D investment for scalability.
**Nanoparticle Stability**	Stability depends on size, concentration, and environmental factors.	Improved stability with “spiky” architectures for targeted delivery.	Stability influenced by lipid/polymer coatings and storage conditions.
**References**	[116]	[117]	[118]

**Table 7:** Comparative analysis of various engineered nanoparticles, including gold, silver, titanium oxide, zinc oxide, silicon oxide, carbon nanotubes, and graphene quantum dots, highlighting their effects on DNA methylation, size and shape dependencies, toxicity, and key study findings.

Aspect	Gold Nanoparticles (AuNPs)	Silver Nanoparticles (AgNPs)	Titanium Oxide (TiO_2_)	Zinc Oxide (ZnO)	Silicon Oxide (SiO_2_)	Single-Walled Carbon Nanotubes (SWCNTs)	Multi-Walled Carbon Nanotubes (MWCNTs)	Graphene Quantum Dots (GQDs)
Key Study Models	Human keratinocytes (HaCaT), embryonic kidney cells, colorectal cancer cells [126]	Pregnant mice (animal model 1); human keratinocytes [127].	HaCaT cells, lung fibroblast cells (MRC5) [128].	MRC5 cells, mouse lung models [129].	Human keratinocytes (HaCaT) [130]	Mouse models, lung cells [131].	Mouse models, lung cells [132].	Limited studies in vitro, exploratory cancer models [133].
Primary Effect	Induces hypomethylation of Alu elements, decreases global methylation.	Alters methylation patterns in placentas and keratinocytes; decreases methylation.	Decreases global methylation and suppresses methyltransferase activity.	Reduces global DNA methylation levels and disrupts ROS balance.	Alters methylation of PARP-1 promoter and decreases methyltransferase levels.	Modulates ATM promoter methylation and influences oxidative stress.	Modulates ATM promoter methylation and influences oxidative stress.	Potential to alter DNA methylation in tumor suppressor genes (e.g., CDKN2A).
Size Dependency	5 nm, 60 nm, and 250 nm sizes studied; 60 nm most potent; medium sizes (e.g., 60 nm) show the highest potency for epigenetic changes.	Smaller particles (<10 nm) are more toxic and disruptive to DNA methylation.	Smaller particles (<100 nm) induce stronger DNA hypomethylation.	Smaller sizes (<50 nm) enhance DNA methylation reduction and methyltransferase inhibition.	Smaller particles (<30 nm) show stronger promoter methylation effects with relatively low toxicity.	Shorter CNTs are more toxic due to easier cellular uptake, DNA damage, and oxidative stress, while functionalization (e.g., carboxylation) further increases toxicity.	Longer MWCNTs cause granulomas and fibrosis due to their asbestos-like structure, whereas shorter MWCNTs mainly induce inflammation and oxidative stress.	Size-dependent changes in fluorescence for methylation detected.
Toxicity	ROS-induced cellular damage observed in keratinocytes.	Cellular damage from placental methylation alterations observed.	Toxicity linked to reduced methylation and oxidative stress.	Similar oxidative stress effects to TiO_2_ but less severe.	ROS-induced changes noted in global methylation levels.	Genotoxicity, pulmonary toxicity.	Genotoxicity, pulmonary toxicity.	No significant toxicity observed in initial studies; potential for safe therapeutic applications.

**Table 8:** Comprehensive comparison of non-coding RNAs (lncRNAs and miRNAs) in cancer, therapeutic nanoparticle delivery systems, and their applications in in vivo and in vitro studies.

Aspect	Long Non-Coding RNAs (lncRNAs)	MicroRNAs (miRNAs)	Nanoparticle Delivery Systems	Therapeutic Potential	Specific Cancer Types Studied	Nanoparticle Advantages	Clinical Trials	Future Challenges	In Vivo Studies	In Vitro Studies	References
Role in Cancer	Regulate gene expression via epigenetic mechanisms and transcriptional control.	Control mRNA translation and degradation; regulate 30% of human genes.	Include lipid-based, polymeric, inorganic, and bio-inspired nanoparticles.	Modify cancer progression via epigenetic regulation and silencing of oncogenes.	Breast, lung, colorectal, gastric, liver, and pancreatic cancers.	High biocompatibility, tunability, and enhanced targeting ability.	ALN-VSP targeting VEGF in patients with liver metastases.	Improve subcellular targeting, reduce systemic toxicity, enhance endosomal escape, and ensure long-term stability.	Exosome-based delivery of lncPTENP1 suppressed bladder cancer proliferation and migration in mice.	AuNPs silenced MALAT1 in lung cancer cells, reducing cell growth.	[145]
Mechanisms	Epigenetic regulation (e.g., DNA methylation, histone modification).	Modulate CpG island methylation and histone modification.	Enhance delivery efficiency for siRNA, miRNA, and lncRNA; target specific cells.	Delivery systems allow precise targeting and lower systemic toxicity.	Lung, breast, glioblastoma, ovarian, and gastric cancers.	Versatility in size, shape, and imaging compatibility for tracking.	Phase I trials of siRNA lipid nanoparticles in liver cancer.	Identify optimal nanoparticle designs for specific tissues and improve nucleic acid stabilization.	Biomimetic NPs successfully targeted lncRNA expression in animal tumor models.	Polymeric NPs silenced lncRNAs and oncogenes in breast and ovarian cancer cells.	[146,147,148]
Therapeutic Strategies	Target overexpressed lncRNAs or restore downregulated ones.	Block oncogenic miRNAs or restore tumor-suppressive miRNAs.	Lipid-based NPs deliver siRNA and mRNA with high efficiency (e.g., Moderna mRNA-1273).	Functional silencing of MALAT1 and other oncogenic ncRNAs.	Colon adenocarcinoma, ovarian, and GI cancers.	Low toxicity and high targeting potential.	Minimal adverse effects observed in early human trials.	Understanding lncRNA subcellular dynamics and overcoming delivery barriers.	siRNA-loaded lipid NPs demonstrated tumor growth inhibition in mice.	Lipid NPs facilitated lncRNA-targeting siRNA in breast and ovarian cancer cells.	[149,150,151]

## Abbreviations

**Abbreviation**	**Full Form**
nsPEFs	Nanosecond Pulsed Electric Fields
HIFU	High-Intensity Focused Ultrasound
ROS	Reactive Oxygen Species
ICD	Immunogenic Cell Death
PDT	Photodynamic Therapy
PTT	Photothermal Therapy
NPs	Nanoparticles
MRI	Magnetic Resonance Imaging
CT	Computed Tomography
SPECT	Single Photon Emission Computed Tomography
PET	Positron Emission Tomography
Y-90	Yttrium-90
